# Application Perspective on Cybersecurity Testbed for Industrial Control Systems

**DOI:** 10.3390/s21238119

**Published:** 2021-12-04

**Authors:** Ondrej Pospisil, Petr Blazek, Karel Kuchar, Radek Fujdiak, Jiri Misurec

**Affiliations:** Department of Telecommunications, Faculty of Electrical Engineering and Communication, Brno University of Technology, Technicka 12, 61600 Brno, Czech Republic; xpospi89@vut.cz (O.P.); blazekpetr@vut.cz (P.B.); xkucha24@vut.cz (K.K.); misurec@vut.cz (J.M.)

**Keywords:** testbed, SCADA, cybersecurity, PLC, HMI, industrial control system (ICS), OT

## Abstract

In recent years, the Industry 4.0 paradigm has accelerated the digitalization process of the industry, and it slowly diminishes the line between information technologies (IT) and operational technologies (OT). Among the advantages, this brings up the convergence issue between IT and OT, especially in the cybersecurity-related topics, including new attack vectors, threats, security imperfections, and much more. This cause raised new topics for methods focused on protecting the industrial infrastructure, including monitoring and detection systems, which should help overcome these new challenges. However, those methods require high quality and a large number of datasets with different conditions to adapt to the specific systems effectively. Unfortunately, revealing field factory setups and infrastructure would be costly and challenging due to the privacy and sensitivity causes. From the lack of data emerges the new topic of industrial testbeds, including sub-real physical laboratory environments, virtual factories, honeynets, honeypots, and other areas, which helps to deliver sufficient datasets for mentioned research and development. This paper summarizes related works in the area of industrial testbeds. Moreover, it describes best practices and lessons learned for assembling physical, simulated, virtual, and hybrid testbeds. Additionally, a comparison of the essential parameters of those testbeds is presented. Finally, the findings and provided information reveal research and development challenges, which must be surpassed.

## 1. Introduction

In recent years, the Industry 4.0 paradigm has been an emerging and hot topic. Many call it “a new industrial revolution”, and they are not far from the truth. Tremendous advances in communication, information, and operation technologies allowed new approaches in the industrial chain to rise. These concepts are industrial internet of things, big data analytics, cloud computing, horizontal and vertical integration, automated robotics and human–machine interactions, augmented reality, additive manufacturing, advanced simulations, virtual manufacturing, and factory twins technologies [1,2,3,4]. Industry 4.0 truly brings up possibilities earlier unthinkable, possibilities with indisputable advantages to the industry. However, it also comes with many challenges which must be surpassed—cybersecurity being crucial. Massive digitalization of industrial processes, growing convergence between information technologies (IT) and operational technologies (OT), the increasing interconnectivity of the different systems are the main reasons for cybersecurity being a challenge [5,6,7,8,9,10,11]. For example, cyber-attacks are a significant incident. When very sophisticated attacks are currently emerging, especially the malware type, which targets these control systems, they are very challenging to detect and prevent, especially zero-day attacks and specialized rootkits. In addition, standard software, and hardware are also present in industrial control system (ICS) networks, which introduces additional potential vulnerabilities. Analyses in this area best demonstrate the extent of potential vulnerabilities. For example, according to a Kaspersky study [12], remote access work in industry increased by 53% during the pandemic. This remote access for industry increases the potential for cyber-attacks, and a reason for this is that remote access can link to the traditional IT infrastructure. Further, according to Asghar et al. [13], only two vulnerabilities in industrial control systems were published in 1997. In 2010, there were already 19 vulnerabilities, and in 2015 there was a significant increase, with 189 different vulnerabilities already reported. We can also see the annual increase in cybercrime in the area of ICS from Kaspersky [12,14,15], from surveys we can see that the trend of incidents in this area is increasing every year, with 415 different vulnerabilities detected by the company in 2018 and 509 different vulnerabilities in 2019.

These vulnerabilities can lead to unimaginable and catastrophic consequences in the ICS, such as an uncontrollable explosion of nuclear power plants or a nationwide blackout. As a result, ICS vulnerabilities can seriously affect industrial production, life, and property safety in our daily lives. Several attacks with significant impacts have already been carried out in recent years. For example, in 2013, an attack with an economic impact was recorded against a German steel mill, where attackers disabled the entire production process and caused significant damage [16]. Other significant attacks have been on the Ukrainian power grid, with the first happening in 2015, cutting off electricity to a quarter of a million households [17]. A year later, a second attack on Ukraine’s power grid took place, which was quite similar and, again, cut off power to almost a quarter of a million customers [18]. Attacks on industrial production can also have significant impacts, especially economic ones, if the attacking parties may be competing companies. Therefore, it is necessary to look beyond the energy sector and more to the manufacturing industry and other parts of the industrial network.

For these reasons, it is necessary to have new intrusion detection schemes for ICS networks of both the process control and network communication levels. For this purpose, the use of machine learning algorithms seems to be appropriate at present [19,20,21,22,23]. However, there is a problem with available datasets suitable for learning, training, and testing. Thus, most researchers are currently forced to build their test environments to simulate the different parts they need.

A testbed (test environment) is a platform that simulates real-world activities under supervision. Testbeds are mainly used for experimental activities and to verify the results of these activities. These activities should also be replicable. Due to the need to generate data from different industrial scenarios, it is necessary to create custom testbeds, especially as mentioned before, due to the low availability of current datasets. Furthermore, it is also necessary to test individual incidents within the operation of each scenario, and this is not possible in the actual operation of individual networks due to the economic impact on the company. Therefore, it is essential to build custom test environments based on real solutions and then test communications and various incidents in these environments to balance the datasets. Finally, it is also advisable to use test environments to test detection methods in real-time. Test environments are therefore a crucial component in ICS security research and are particularly suited to these activities:

- Research into attack technologies and, in particular, possible defences against these attacks and assess vulnerabilities, discovering exploits within a particular software or hardware test environment.
- Research in anomaly detection. This category includes activities such as dataset generation, threat detection, selection of appropriate models and algorithms for detection and optimization of detection solutions.
- Optimization through functional tests. This part includes testing the reliability of individual components and optimizing for the actual operation, the impact of changes on hardware or software or the entire area, and using this information to create a performance analysis. Functional tests can be also helpful in the optimization of an incident detection system.

Individual test environments can provide information on essential security parts of industrial networks. Based on this, datasets can be created for an intrusion detection system (IDS) based on network communication or for an IDS that focuses on process information.

In this context, the contribution of this article is mainly in the following directions:

- We provide the current status of testbeds in industrial control systems, focusing on all sectors outside the power industry.
- We have created a summary of the parameters of each testbed in this area.
- We described our experience with creating physical, simulated, virtual, and hybrid testbeds.
- From the summary of the testbeds, we visualized the percentage representation of each parameter, allowing us to define challenges for further research in this area.

The paper is structured as follows. In the introduction, we discuss why it is crucial to create testbeds for industrial networks, and we provide background to testbeds. We also discuss the current state of the art in the field of testbeds, where we provide our methodology and summary tables of individual parameters of industrial testbeds. Section 3 describes our approach to testbed creation and presents our experience in creating physical, simulated, virtual, and hybrid testbeds. We also discuss what we have learned through construction and provide our plans with individual testbeds. Section 4 presents a visualization of each parameter from the summary tables we discuss, providing challenges for potential further research in this area.

## 2. Related Work

In our article, we created a comparative study of the current state in the field of industrial testbeds to improve our knowledge. This comparison helps to understand industrial testbeds’ current situation in process automation. We focused only on the category of process automation (where the programmable logic controller (PLC) is the primary controller), and we deliberately omitted the testbeds from the energy sector in the comparison. To find out the current state of the survey articles on this issue, we systematically searched for individual articles. We used the specific set of keywords ((testbed OR testbed) AND survey AND (ics OR “Industrial control system*”)) to get as many relevant articles as possible. We used this term in the Scopus database, where we obtained seven results (comparable results were also in the Web of Science database). After researching the individual articles, we were left with two articles [24,25] dealing with our area of interest. We then added survey articles, which we got through references from articles on testbeds and from unstructured searches via google scholar. Thanks to that, we found another three relevant articles [26,27,28].

In the article by Holm et al. [24], the authors focus on exploring which testbeds are designed for scientific research. What are the research goals of the current testbeds? How are the ICS components implemented in these testbeds, and how do they handle the requirements? Their comparison focused mainly on these parameters: the objective of testbeds, components in testbeds, implementation of these components (virtualization, emulation, simulation, and hardware), and fidelity. They also focused on exploring possibilities of using testbed while describing the following categories: vulnerability analysis, education, tests of defence mechanisms, power system control tests, performance analysis, creation of standards, honeynet, impact analysis, test robustness, tests in general, and threat analysis. These categories can be used for further research. The article is from 2015 and is therefore relatively outdated in the field of testbed summary. However, it still provides a good starting point for selecting parameters for comparing current testbeds. From the article, it is also clear that the central area of application of testbeds lies in cybersecurity research and education.

Cintuglu et al. [26] deal with the comparison of smart grid testbeds and they omit process automation testbeds. In their comparison, they focused on the following parameters of testbeds: targeted research area, covered smart grid domains, test platform type (such as simulator, hardware, real-time simulator), communication protocols, technology (Ethernet, RS232, Wi-Fi), network type, and connection type (wired or wireless). The article is of an older date (2016) and therefore does not include the latest testbeds. In contrast to our comparison, it focuses primarily on smart grid testbeds. The article has an excellent methodology for comparing parameters; we also used some of the parameters in our comparison.

Qassim et al. [27] deal mainly with the description of testbed approaches (such as physical, simulated, virtual, hybrid). The article also focused on essential parameters of testbeds: fidelity, repeatability, accuracy, safety, cost-effectiveness, reliability, and scalability. They described how these parameters are evaluated from the point of view of the testbed approach. This article does not summarize testbeds, and it only recommends how to assess properties within the testbed approach. From this summary; we used fidelity assessments within the testbed approach.

Geng et al. [25] focused on the analysis of implementation methods for ICS components in testbeds. They described their classification testbed approach. They classified testbeds as physical simulation testbeds, software simulation testbeds, semi-physical simulation testbeds, and virtualized testbeds. The article also described eleven possible application scenarios for testbeds, which are different from the scenarios described by Holm et al. [24]. They divided the scenarios into the following categories: attack technology research, defence technology research, vulnerability assessment, education, security control system development, functional test, supervisory control and data acquisition (SCADA) training, security verification, forensic analysis, safety standards development, and cyber security competition. The article does not compare testbeds, it describes only the evaluation options, approach and defines possible application scenarios.

Ani et al. [28] described the methodology very thoroughly for evaluating testbeds. The methodology focused mainly on the fidelity of the created testbed, and authors emphasized the essential parameters when creating testbeds with much fidelity. They presented a very good summary of the features before building a testbed. When comparing the testbeds in the table, they focused on the following properties: objective, approach, landscape/coverage, credibility requirements (such as fidelity, flexibility, openness, adaptability, safety), and evaluation/validation. In the overview of testbed comparisons, the article focuses more on energy testbeds. It describes which parameters to focus on and what is essential for testbeds in security testing to make their behaviour as reliable as possible. In the overview of testbed comparisons, the article focuses more on energy testbeds. In contrast, we focus mainly on testbeds within the process automation. When creating the evaluation methodology for our article, we followed some recommendations for comparative parameters. For us, this parameter is relatively essential since we are interested in network communication in the testbed.

The main approaches and parameters of unique articles were described in the text. In the mentioned articles, they did not always approach the same evaluation and description of individual parameters. In our paper, we also approached some parameters in our way; the description follows next.

Above, we described what the survey articles in industrial testbeds deal with. We described the main focus of the mentioned articles and what information we used from these articles. One of the main reasons for doing another survey is that the articles [24,26,27] are outdated do not contain newer testbeds. Another reason is that these articles focus more on power system testbeds. We focused only on process automation and deliberately avoided power systems because few articles are devoted to this area. Furthermore, when comparing testbeds, we also focused on their properties, which can be a good measure for the use of testbeds in creating new datasets for intrusion detection systems working with machine learning. A significant parameter that they deal with in only one survey [26] is the testbed using cloud services. It is vital to include this parameter in the comparison due to one of the directions of current research in the field of industrial control networks, which is infrastructure as a service (IaaS) offered by cloud providers [29].

### Methodology

Thanks to a previous comparison of articles on testbed parameters, we found out which parameters to focus on and how to approach them. In individual articles, authors did not always approach the evaluation and description of individual parameters in the same way. In our paper, we have optimized the approach to evaluation and category definition for some parameters. We divided the individual categories mainly from the point of view of cybersecurity and the possibility of traffic analysis. That is why we chose the given categories. Moreover, they are essential for us from the point of view of data collection, analysis, subsequent processing of this data and creation of datasets which can then be used for model training and subsequent detection of anomalies in industrial operation. Description of parameters, as we approached them, follows below.

For each article, we will compare parameters of individual testbeds and show these parameters in Table 1 and Table 2. Subsequently, we will describe the individual parameters of the table here. The table is sorted according to the year of publication of individual articles. Further, the table will contain parameters like the name of the institution and reference to the article. In the summary table, we will also consider whether the testbed used a SCADA system. This means if it uses any specialized software for SCADA systems that can remotely control the device, which is essential information from a cybersecurity perspective. Other elements will be described in more detail in the following text:

**Scenarios:** Here the individual scenarios are listed, ordered according to a created testbed and operation it can perform. We will describe the individual scenarios and assign them a suitable icon to save space in the table. We will describe individual scenarios and assign them a suitable icon to save space in the table.

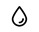
**Water treatment:** All scenarios that are linked with water treatment are included in this section, for example water storage, water tower, water distribution networks, wastewater treatment, water reservoir, and others.
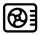
**Cooling and ventilation:** This category includes cooling and ventilation systems such as industrial blowers, and cooling systems for supercomputers.
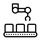
**Production line and material processing:** In this category, we have included all industrial processes that have something in common with the conveyor belt, processes such as robotic assembly, conveyor belt, steel rolling, smart manufacturing, and others.
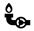
**Pipeline transport:** In this category there are scenarios trying to mimic some part of the pipeline transport, such as the gas pipeline.
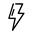
**Energy industry:** Although we have included in our work only process testbeds with an intentional avoidance of testbeds that simulate the energy industry, some testbeds that are composed of several different parts also contain an energy component. That is why they are marked; these are, for example, smart grid networks or power transmission.
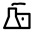
**Industrial plant:** In this category, we have included thermal plants, chemical processes, and more.
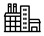
**General model of industrial networks:** Here, we have included testbeds that are built as a general model of industrial networks, these testbeds are not specialized.
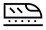
**Transport:** Any transport within an industry such as rail transit.

**Category of testbeds:** In this part, we followed the already proven division of testbeds from previous works.

- Physical testbed—All testbed parts are built on physical hardware and try to get as close as possible to real solutions.
- Simulation—The testbed is created using simulation, so the individual parts are simulated in software.
- Virtual testbed—The individual parts are created using virtualized devices that can run on separate hardware as emulations.
- Hybrid testbeds—Such testbeds are a combination of previous solutions.

In individual articles, a combined solution (hybrid testbeds) is often used; therefore, the table also evaluates and describes layers on which is an approach used.

**Fidelity:** Our approach for this parameter was different from other articles; thus we have created our own evaluation, which will be explained below. We focused on whether the testbeds are based on real systems. That means whether the authors tried to follow parameters and functionalities based on discussions with experts or their own experience when creating the testbeds. We also focused on whether these testbeds are based on standards for the area. Another parameter that provides essential information about fidelity is how the testbed is constructed, meaning whether it is a physical replica (has the highest fidelity score), simulation (has the lowest fidelity score), virtual (average score), or hybrid (depending on the design).

We evaluated the testbeds as follows: each testbed can get a maximum of 5 points. Depending on the approach to building the testbed, the physical testbed can get 3 points, virtual/hybrid 2 points, and the simulation 1 point. Each testbed can also receive a point for trying to imitate credibility according to studies and discussions with experts and another point for adhering to standards (the testbed must come close to a real use). The ideal testbed should therefore have 3 + 1 + 1 points.

**Application scenarios:** We have divided application scenarios into four categories inspired by previous researches. Subsequently, we assigned subcategories to each category and defined these subcategories.

- Cybersecurity (vulnerability analysis/academic security research)
  − Research into attack technologies and research into possible mitigations.
  − Vulnerability assessment, discovery of exploits on certain testbed software or hardware.
  − Development of a security management system that is more secure and better deals with possible threats.
  − Anomaly detection.
  − Forensic analysis.
  − Environment for capture the flag (CTF).
  − Honeypot/honeynet.
- Education
  − General knowledge of ICS.
  − Education of students in the branch.
  − Programming of industrial equipment.
  − Training of SCADA operators.
- Functional tests
  − Reliability test of individual parts and equipment.
  − Optimization of industrial operation.
  − Impact of hardware (HW) and software changes on industrial operation.
  − Performance analysis.
- Development of standards
  − Research and development of safety and cybersecurity standards that are in line with the industry.

**Figure 1:**
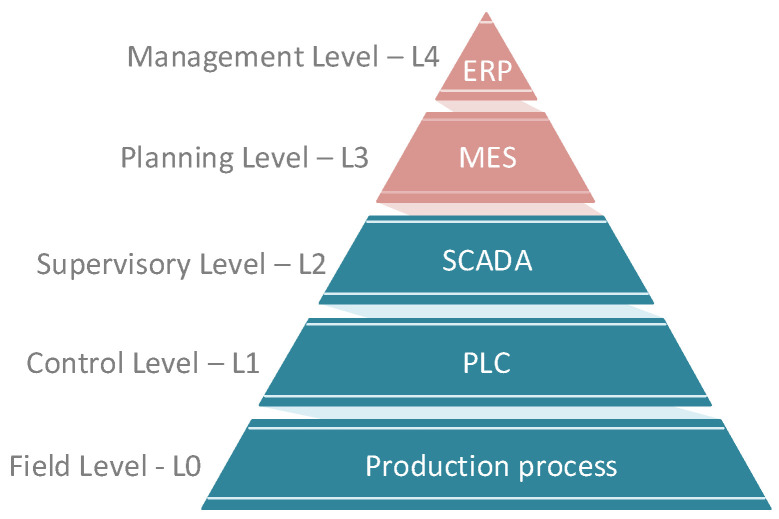
The automation pyramid based on the ANSI/ISA-95 (ISO 62264) model.

**Levels:** This parameter is based on the scheme of the automation pyramid consisting of five layers. Most testbeds contain only L0–L2, as indicated in Figure 1. Higher levels (red) are not included in the testbed comparison.

- Level 0—Field Level: It is the level where the production process takes place. At this level, work all parts that move physical production. Devices like actuators, sensors, intelligent electronic devices work here. There are also process communication protocols that communicate with Level 1.
- Level 1—Control Level: Programmable logic controllers (PLCs) work on this layer. The whole distributed control system (DCS) works on this layer if it is a more extensive operation. The control process means the analysis of Level 0 inputs and outputs is in progress at this point.
- Level 2—Supervisory Level: SCADA systems work on this layer. These levels are used for remote monitoring and control of lower ones.
- Level 3—Planing Level: The manufacturing execution system (MES) works on this layer, monitoring the entire production process from raw materials to the finished product.
- Level 4—Enterprise Level: This is a management level that uses an integrated management system of the company, which is called enterprise resource planning (ERP). Here the company’s management can see and manage their operations.

The summary table also indicates, in several colours, how the implementation is approached on each layer, i.e., which approach has been chosen:

- The layer is based on physical hardware (marked in blue 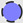)—it must satisfy the condition that there is physical industrial hardware in the layer that is dedicated to the operations.
- The layer is based on emulation (marked in yellow 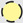)—it must satisfy the condition that other devices emulate individual devices to replace real devices.
- The layer is based on simulation (marked in green 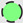)—it must satisfy the condition that the software simulates the individual parts. Virtualization also falls into this category.
- The layer is based on a hybrid solution (combination of colours)—it is a combination of different approaches.

**Protocols:** When selecting essential protocols for the industry, we focused on the most popular protocols these days and only on those based on industrial Ethernet. The reason is that according to a survey by HMS Networks [61], the share of Fieldbus decreases every year (28%), and the share of industrial Ethernet increases (65%). At present, Profinet has the largest market share (18%), followed by Ethernet/IP (17%), EtherCAT (8%), Modbus-TCP (5%), Powerlink (4%), and others. In our comparison, we considered only the protocols: Ethernet/IP, Profinet, EtherCAT, and Modbus-TCP. Any of the mentioned testbeds did not use the Powerlink protocol, so we excluded it from the comparison. Instead, we added Siemens’ proprietary S7 protocol to the comparison due to its large market share in the European market.

**Industrial components:** In this comparison, we focused on the essential components in industrial networks that are important for testbeds due to the possibility of obtaining data from the operation. These are elements such as PLC, Human–Machine Interface (HMI), Master Terminal Unit (MTU), Remote Terminal Unit (RTU), Historian, and Engineering Workstation. In this comparison we also studied whether the testbed uses SCADA systems in its implementation.

**Characteristics of testbeds:** The last thing we compared were the properties, characteristics, or focus of individual testbeds. Where we focused on what the testbeds were created for their characteristics and what they were used for.

- **Data analysis:** We included testbeds in this category, which were in some way focused on working with data: generation, monitoring, logging, and dataset creation.
- **Flexibility/adaptability:** An attribute that tells whether a testbed can redefine its primary usage to another usage scenario. That is if the testbed can be used for more use cases. We focused primarily on whether this property is indicated or emphasized in some way in individual testbeds.
- **Scalability/Extensibility:** It is possible to easily expand the testbed in terms of functionality and by adding components (sensors, actuators, etc.).
- **Cybersecurity solutions:** Here, we focus on whether the testbed is created to test, improve, or create cybersecurity solutions.
- **Cloud solutions:** We decided to include information on whether the cloud can be used in the testbed, especially the infrastructure as a service (IaaS), due to the growing interest in this area [29,62,63].

## 3. Our Approach—Lesson Learned

In this section, we will describe our approach and experience with the creation of individual industrial testbeds. In our research activities in the field of industrial cybersecurity, we have created and designed testbeds falling into all categories; i.e., physical testbeds (testbeds purely based on industrial HW), hybrid (emulated testbeds with a combination of elements from industry) and emulated testbeds, virtual testbeds (testbed based on a virtualization platform), and simulation testbeds (testbed built as simulations in software). We will describe how we approached the individual testbeds, what we have learned, what needs to be changed, and what we plan to add to the individual testbeds after the experience with creating them. All of our testbeds were created in the university laboratory and are placed there and used for data collection.

### 3.1. Experience with Physical Testbeds

Physical testbeds are based on physical industrial hardware. These testbeds give us the most information about the real behaviour of the device in real operation. We currently have a ready-made testbed for general testing and interception of communications on components from a single company. We also created a physical testbed that simulates the behaviour of a wastewater treatment plant. Lastly, we have designed a testbed for the simulation of the brewery, which we are currently creating after previous experience with building testbeds.

#### 3.1.1. Testbed for Industrial Motor Control System

**Figure 2:**
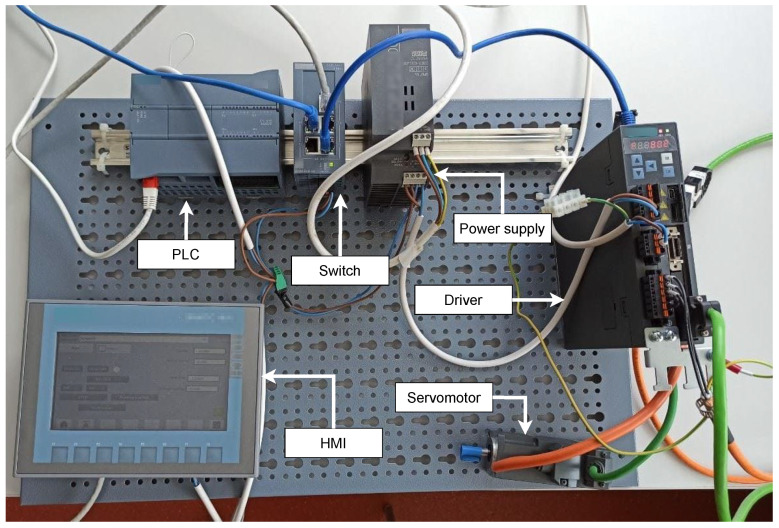
Testbed for industrial motor control system.

This testbed is based on Siemens components. All components inside the testbed are products of this company. The main goal of creating this testbed was the possibility of access to communication data within Siemens communication, based on which datasets would be created. As seen from the testbed survey, a minimum of testbeds deal with this topic, and no datasets with this communication are available. The physical testbed can be seen in Figure 2. We created this testbed in the first quarter of 2021. It is a simple testbed by design that can be created in a few days.

##### Diagram of Industrial Motor Control System Testbed

**Figure 3:**
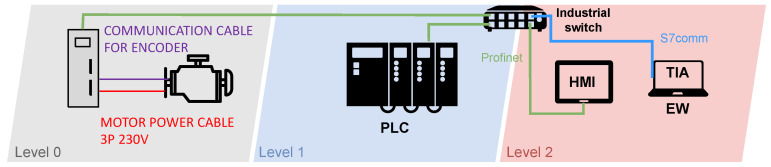
Diagram of industrial motor control system testbed.

The diagram of the testbed can be seen in Figure 3. The diagram is divided into three parts based on the automation pyramid. There is a production process (field level) in the first grey part with two components. Here we used a servo motor together with a frequency converter. This frequency converter can also be called a driver that communicates with the motor using a particular cable. The driver is connected to an industrial switch in order to receive instructions and thus communicate with the PLC. The PLC, which controls the entire testbed, works on the blue layer (control level). The last red layer (supervisory level) contains the HMI for process control and motor operation. On this layer is also the work engineering workstation (EW). It is a station uploading new programmes to individual devices or updating these devices. This station can also be used to monitor the network, using a specific software it contains. All components communicate with each other via an industrial switch.

##### Components

Table 3 lists individual components of the testbed. It is a listing of hardware (devices and components), software, and protocols.

##### Scenario

The testbed focused mainly on the S7 communication protocol. The scenario is motor control via HMI and sending specific motor commands; commands such as starting and stopping the engine. It is possible to set the exact speed and send this information to the engine. It is also possible to change the direction of rotation of the motor. The motor speed and the current motor position can be supervised on the HMI.

##### The Purpose of the Testbed

We built this testbed primarily for the possibility of obtaining data for datasets from an environment that is based on proprietary communication. This is Siemens’ communication and their S7 protocol, which is based on the Profinet protocol. The data obtained will be used for anomaly detection tools.

##### Lesson Learned, Future Work, and Limitations

Thanks to the building of this testbed, we were able to perform a deep dive into the communication problem of Siemens’ proprietary S7 protocol. The testbed allows us to capture communication and analyze this communication. We can use these data to lay the foundation for a detection system in these proprietary systems. In the future, we would like to expand the testbed with other Siemens components. The goal is also to make communication with this testbed using the SCADA system. We would also like to use the new functionality of the TIA portal version 17 in the testbed, namely the new generation of HMI within WinCC Unified, based on HTML5 and JavaScript web technologies. Finally, it is planned to perform long-term interception of communication in this system with the possibility of input of various anomalies. The main limitation of this testbed is its flexibility, where the testbed is designed for only one purpose, namely motor control.

#### 3.1.2. Wastewater Treatment Testbed

**Figure 4:**
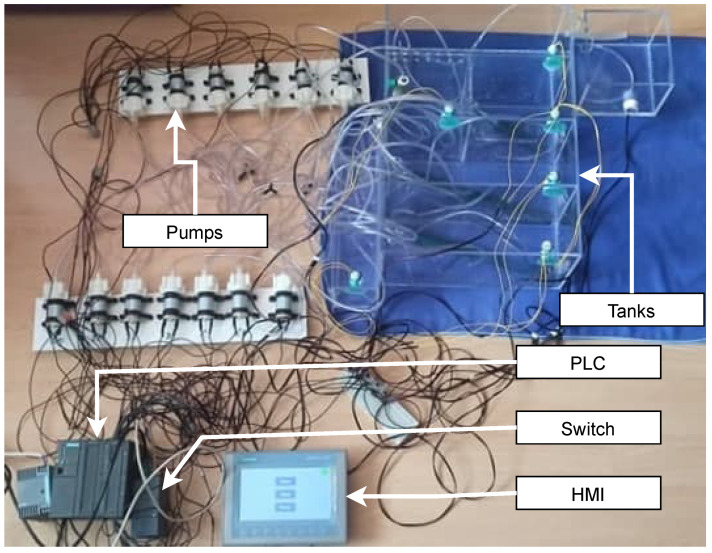
Wastewater treatment testbed.

Wastewater treatment plant testbed tries to get as close as possible to natural treatment plants with sequencing batch reactor (SBR) technology. It must be noted that this testbed was designed according to an actual treatment plant for the village. The main goal of the building of this testbed was the possibility of collecting process data (from pumps and sensors). We chose a wastewater treatment plant because more and more treatment plants (for homes, companies, villages, or larger areas) are controlled and monitored remotely using SCADA systems. Thus, this testbed is used to research cyber security and the possibility of vectors of attacks in this area. The primary goals for us are process data collection and cybersecurity research. The physical testbed can be seen in Figure 4. This testbed we were creating was completed in June 2021 but is still being actively worked on and optimized for operation. As it is a physical testbed, the design process was quite complex and took a lot of time and planning.

##### Diagram of Wastewater Treatment Testbed

**Figure 5:**
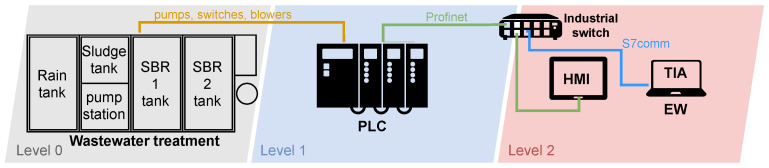
Diagram of wastewater treatment testbed.

A diagram of the wastewater treatment plant is depicted in Figure 5. Again, the scheme is divided according to the industrial pyramid as in the previous case. Layer 0 is the physical wastewater treatment plant. The individual pumps necessary for the plant operation operate, followed by level sensors, sludge sensors, pumping sensors, rainwater sensors, and blowers. A PLC from Siemens operates on layer 1, which controls the entire wastewater treatment process. An engineering workstation operates at layer 2, from which the treatment plant program can be modified, or the operation can be supervised. There is also an HMI on this layer, which allows the operator to control the individual processes of the wastewater treatment plant. An industrial switch again connects the individual components.

##### Components

Table 4 lists individual components of the testbed. It is a listing of hardware (devices and components), software, and protocols.

##### Scenario

The entire wastewater treatment plant can be controlled via HMI. From this interface, the whole process can be started or stopped. The HMI can check or adjust the status of the blower, the status of pumps, and the status of tanks. The HMI can also send a command to drain the entire wastewater treatment plant. On the blower status screen, we can check if the blower is running. Parameters that affect the operation of the blowers, such as the number of aeration and sedimentation cycles and their running time, and the aeration time in the sludge tank, can be set here. The pump status screen displays the status of all ten pumps. It is also possible to control the pumping of sludge into both SBR tanks, set the pumping time from the SBR to the micro sieve tank, and finally, to set the pumping time from the SBR tanks. The tank status screen can be used to check the maximum and minimum levels of the five plant tanks. On the last screen, the drainage of the wastewater treatment plant, the tanks’ status, and the pumps’ status when pumping water can be checked.

The entire usability of the wastewater treatment plant is derived from the HMI control. The whole model consists of seven tanks, ten pumps, and ten sensors. Firstly, the stormwater retention tank pumps water to the pumping station; this happens whenever a minimum level is indicated in the pumping station. Next, the pumping station pumps water to the SBR1 tank. Filling, aeration, and sedimentation are repeated in cycles. After the completion of each cycle, the SBR1 tank pumps water to the micro-screen tank. The sludge is then pumped and aerated. Meanwhile, when the water filling process of the SBR1 tank is completed, water from the pumping tank starts to fill the SBR2 tank, where the same process, as for the SBR1 tank, takes place. After all cycles are completed, the contents of the SBR2 tank are pumped into the microsieve tank. Finally, pumping from the receiving tank to the pumping station occurs, the process proceeds as previously described. The scenario runs automatically in cycles, but it is also possible to control it manually and pause and restart the whole process at the step where it was paused.

##### The Purpose of the Testbed

The testbed is intended primarily for long-term data collection of standard operation, where the collection will focus primarily on process data. The main focus is on controlling data from monitoring devices, currently from HMI. In the next phase, we will focus on data collection from SCADA systems. This data will again be used to create datasets for industrial detection tools.

##### Lesson Learned, Future Work, and Limitations

Thanks to the creation of this testbed, we got to know how real wastewater treatment plants work, and we were able to get more in-depth into the issue of industrial communication, which is based on a real solution. We also used the Siemens S7-300 PLC, which is now replacing the S7-1500 PLC, and we ran across some of its limitations during the design. However, we used this PLC deliberately because it is often present in companies, and it is helpful to be able to test the operation on this PLC as well. Currently, our goal is to implement the device in our SCADA system, which is based on OpenMUC. The plan is to intercept remote communication, which should be implemented using the OPC protocol. We also plan to implement a visualization environment in WinCC Unified for the plant. The limitation of this solution is mainly the lack of simulation of chemical processes, which usually take place in wastewater treatment plants. Thus, process data from this area is missing. This is another opportunity for future improvement of this testbed.

#### 3.1.3. Brewery Testbed

The brewery is one of the typical representatives of industrial production, where the automated process is widely used. It contains several different sensors, switches and valves that provide a large sample of data (various digital and analogue values). At the same time, the process is not very complicated, and it is relatively easy to analyze the communication and assign it to data from individual sensing and control elements. We started creating this testbed between the first quarter and the fourth quarter (cooking process optimization) of 2021. During the implementation, we faced a number of problems such as—design of the cooking process in automated form, selection and completion of suitable components or identification of non-standard conditions.

##### Diagram of Physical Brewery Testbed

**Figure 6:**
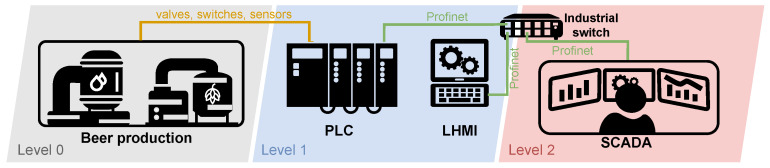
Diagram of physical brewery testbed.

Despite the complexity of the structure of the elements (sensors, switches, valves), it is possible to control the production of a smaller brewery using one PLC unit, as shown in the simplified diagram in Figure 6. The diagram is based on the automation pyramid, where testbed elements are assigned to the corresponding level of the pyramid. In the lowest level 0, sensors and control elements are connected using continuous and state signals to the input/output interfaces of the PLC unit located at the higher level 1. The PLC unit controls the entire production process and mediates communication with the HMI and SCADA display interfaces. The local HMI (LHMI) unit, at level 1, is used for control. It can also be used for control directly at the factory. The unit is connected to the PLC unit via an industrial switch; communication is ensured by the Modbus/TCP application protocol and the reliability of the TCP/IP family of protocols. Furthermore, data from the PLC unit is transferred to a higher level 2 using the same technology as with the LHMI. Here the data are processed and evaluated by the monitoring and control centre (SCADA).

##### Components

The physical testbed of a brewery is composed of many parts listed in Table 5. Individual terminal elements (such as valves and relays) are only listed here in general terms. Any devices compatible with the used PLC can be also used for implementation.

##### Scenario

**Figure 7:**
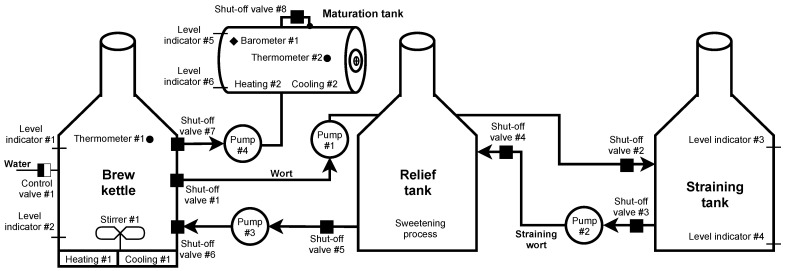
Diagram of physical brewery scenario.

Brewing beer is not a complicated process, but it is necessary to check and follow procedures. In our scenario, the control is provided by the Siemens S7-300 PLC unit. Several sensors and switches are connected to this PLC, informing about the status and automating the entire production process. If processes such as automated dosing and preparation of ingredients (such as shredding, the addition of malt grist, hops, yeast) used mainly in large breweries are omitted, then brewing begins with the mashing process. Mash is a brewing mixture of water and malt grist; mashing is a process in which this mixture is heated to certain technological temperatures. The aim is to divaricate the starches forming a large part of the endosperm into simpler sugars with the help of malt enzymes, activated at specific technological temperatures. The structure of such a scenario is shown in Figure 7.

Mashing takes place in the brew kettle, where the process is monitored by checking the temperature using thermometer #1 and the amount of mash (level indicator #1, #2). At the beginning of the process, control valve #1 is opened, and the required amount of water is added to the malt grist. Subsequently, the temperature is maintained for the required time using heating #1. When the mashing is completed, shut-off valves #1 and #2 are opened up and the pump #1 starts working. The wort created by mashing is pumped to the straining tank, where the straining process occurs. During pumping, the level is monitored by the level indicator #3. After filling, the pump #1 is switched off, and shut-off valves # 1 and # 2 are closed. In the straining tank, the wort (liquid component = sugars and other soluble substances from malt) separates from the malt mortar (solid component; leached malt grist).

Subsequently, the wort is strained into a relief tank; this step is activated by opening the shut-off valves #3 and #4 and starting pump #2. The remaining unwanted substances are removed from the wort in the relief tank so that the brewing process is not contaminated (contamination would affect the resulting taste and smell of the beer). After completing the process, opening shut-off valves #5 and #6 and starting the pump #3, the resulting wort is pumped into the brew kettle.

Pump #3 is switched off after pumping, and shut-off valves #5 and #6 are closed, followed by the opening of control valve #1 and replenishing the wort with the required amount of water. After signalling level indicator #1, control valve #1 is closed, and heating #1 starts. In this phase, the substance is brought to the boiling point, where it is maintained at the desired temperature. Thermometer #1 monitors the temperature at all times. The measured amount of hops is added to the substance at a specific time interval. Hopping takes place two to three times during the brewing process. After the brewing process is completed, the cooling process follows. Heating #1 is switched off, and cooling #1 starts, supported by starting the stirrer #1 for faster cooling. The aim is to cool the wort to fermentation temperature and remove sludge. It is necessary to get rid of these sludges as much as possible. They would adversely affect the fermentation process, causing unnecessary oxidation, and the risk of contamination increases. The stirrer #1 not only provides faster cooling, but the sludge is concentrated in the lower part of the vessel due to the formation of a whirlpool. Therefore, there is no need for a filter unit to remove sludge during the subsequent pumping. After cooling to the desired temperature (monitored thermometer #1), the wort is pumped into maturation tanks. Shut-offs #7 and #8 are open and the pump #4 is running. At the level indicator #2, pump #4 is switched off, and shut-offs #7 and # 8 are closed. During pumping, the level is monitored in the maturation tank using level indicator #5 to prevent tank overfilling. Subsequently, yeast is added to ensure the maturation of the beer.

The last stage of beer production is primary and secondary maturation or fermentation. After adding yeast, the temperature and pressure are monitored in the maturation tank. Constant temperature is one of the most important parameters when beer is matured. The added yeast has the most significant production only in a specific small temperature range (usually between 4 and 8 degrees). If the interval is exceeded, side effects are created that affect the taste and smell of the beer. The temperature is monitored by thermometer #2, and in case of excessive temperature rise/fall, cooling #2 or heating #2 starts. During fermentation, sugars are converted into ethanol and carbon dioxide, which increases the pressure in the maturation tank. In addition to the fact that pressure too high could damage the maturation tank, keeping the pressure value in the range is crucial to avoid oversaturation of the resulting beer. Barometer #1 monitors the control and possible release of pressure.

##### The Purpose of the Testbed

The testbed was designed to simulate various terminal elements such as switches (relays), binary valves, sensors, weight, and temperature. All these elements are relatively simple to implement in a simulated environment. Only in a complex physical environment/scenario, their coordination can be observed in cases where, e.g., one or more elements will generate non-standard conditions (such as data and signals), for instance, valve failure, the temperature outside a defined interval and more. Thanks to precise brewing procedures, it is possible to observe the influence of these non-standard conditions during the whole process, analyze them, and draw clear conclusions. Due to its complexity, the testbed is also suitable for long-term data collection; this data can be used as an input for further learning, e.g., neural networks or artificial intelligence.

##### Lesson Learned, Future Work, and Limitations

By implementing a physical testbed for beer production, we verified a complex process consisting of different end devices working with one Siemens S7-300 PLC unit. Currently, the testbed is primarily controlled by an HMI interface. The SCADA openMUC is composed of a display unit (receives and visualizes data from the PLC unit) but it does not provide full support for the SCADA unit. Following this observation, our primary goal with this testbed is to implement all elements in the openMUC so that it provides all parts of the SCADA system. Furthermore, a detailed analysis of the Profinet protocol communication between the PLC unit and openMUC will be performed. The main limitation of this testbed is its flexibility, where the testbed is designed for only one purpose, namely beer production. Although the testbed allows the brewing of different types of beer, their impact is minimal on communications and thus more extensive data variability.

### 3.2. Experience with Virtual Testbeds

#### 3.2.1. Honeypot Testbed

With automation and remote control in the industrial sector, attacks on these systems are becoming more frequent. However, analyzing such an attack is not easy. While dealing with an attack on a system, the key is not to identify and analyze it but to prevent its spread and progress. In the industrial sector, devices used are more proprietary, so simulation is not as simple as in classical IT networks. Based on this, we have a procedure where a so-called honeypot will be deployed to the Internet, pretending to be an actual industrial device and performing logging in the attack, which will help us analyze and document the attack.

We implemented this test environment throughout 2021. The testbed is still under development and its implementation is quite demanding. Virtualizing elements so that an attacker does not recognize that the device is not real is quite challenging. In the future, we anticipate expansion with additional virtualized devices, communication protocols, and network infrastructure elements.

##### Diagram of Honeypot Testbed

**Figure 8:**
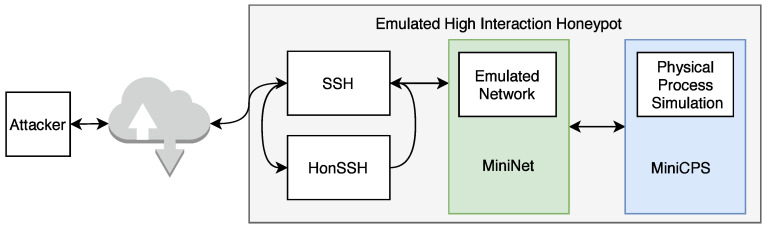
Diagram of honeypot testbed.

A new highly interactive honeypot was developed for the possible analysis of network attacks on industrial devices and networks. The honeypot simulates actual services and ICS communication protocols to facilitate a network that can be expanded based on the developer’s needs. The following Figure 8 illustrates the basic structure of the new high-interaction honeypot.

The entire system uses multiple parts: (i) MiniCPS, which simulates physical processes, (ii) MiniNet to simulate network topology, (iii) HonSSH as a solution for recording bash activity, and (iv) containerization using Docker isolates the attacker from the components of the host machine. The goal was to build on the security research toolkit for the industrial control system security MiniCPS. The honeypot will emulate protocol services running on the emulated network MiniNet topology, connecting it to the main interface of the virtual private server with static routing through a virtual switch (also emulated by MiniNet). To access emulated network, SSH port 22 is exposed to the Internet. Moreover, HonSSH listens to all incoming traffic to this port. The attacker is forwarded to the Docker container that exposes port 2222 to the main VPS interface through port forwarding. From the Docker container, the attacker presents processes running from MiniCPS and the Ubuntu kernel. If the container is broken, it is easily recoverable from a previously created container image.

##### Components

The honeypot is built on a vitalized platform in order to depend on the required performance but not on specific HW components. Table 6 below lists all the software and protocols used. The core of the whole system is the Ubuntu 10 buster vitalized operating system (Ubuntu VPS) on which the individual applications—HonSSH, MiniNet, MiniCPS, Docker, Suricata, and Grafana—are installed. The combination of these applications creates a complex system that allows analyzing incoming attacks from the Internet in a secure mode.

Two libraries enabling Modbus and Ethernet/IP protocol communication are used to virtualize industrial system elements. The Modbus protocol is simulated using the pymodbus library. This library is written in Python and provides a full Modbus protocol implementation by utilizing twisted for the core of its asynchronous communications. No additional dependencies are required for this library to function. CPPPO implements a subset of the Ethernet/IP protocol utilized by some industrial control equipment; it is also written in Python.

##### Scenario

**Figure 9:**
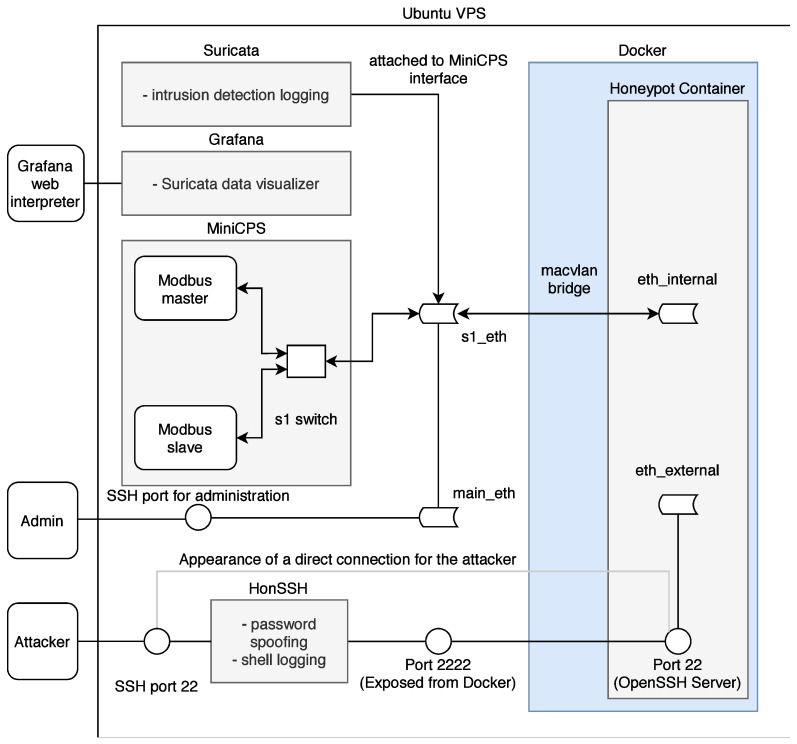
Diagram of honeypot scenario.

The honeypot scenario is divided into two parts according to simulated protocols listed in Table 6. The interfaces, on which the processes are running and the simulated topology, are visible to the attacker. Therefore, the intruder can capture the packets generated by these processes. Moreover, the attackers can plant their packets, on which the master node in the topology will acknowledge these requests. The diagram in Figure 9 shows the entire honeypot structure.

MiniCPS provides the simulation of industrial network entities, where the library directly includes SWaT (water treatment), which was used for the next implementation. A temperature sensor was selected from this library, which uses the random function random.uniform (a, b) to simulate the value of N with the parameters a< = N< = b for a< = b and b< = N< = a for b<a. This variable random value N from the interval <a, b> subsequently served for both as an output from the temperature sensor communicating with the protocol for the given scenario.

Containerization is an integral part of separating an attacker from the part of the system where he should not have access. At the beginning of the implementation of the honeypot, a function called chroot or chroot jail was used, which forms a separate root directory. An attacker from this directory does not have access to the rest of the Linux system and commands through which he could damage the operation of the entire honeypot.

The SSH protocol was built on the same principle in the first design, where all connections from the attacker were to be chroot-jailed using OpenSSH´s ChrootDirectory feature. Nevertheless, after further examination, this objective was abandoned due to insufficient control over the entire function and thus the impossibility of ensuring the required security.

Instead of ChrootDirection, a more suitable and simpler Docker containerization together with the macvlan bridge function was chosen. Docker is directly designed to redirect traffic through the main interface of the honeypot (e.g., eth0 on Linux) to the implemented container using the MAC address. For the container and the attacker, it looks like the container is connected directly to the Internet.

Another advantage of containerization over chroot is the simpler multi-level root filesystem namespace. The namespace allows for a dedicated network container stack. The next part of the UTS settings is used to set the hostname and domain, visible to running processes in the given namespace. Additionally, the last important IPC setting provides separation of shared memory segments.

Since the honeypot is connected directly to the Internet and the SSH connection via port 22 is redirected via HonSSH to port 2222 to Docker, it was necessary to create a connection for the administrator to access the private part of the honeypot and not just the public part. For this reason, the sshd_config configuration file was modified so that in the case of public-key authentication, the connection is established directly to the Ubuntu VPS.

##### The Purpose of the Testbed

With the increasing advent of automation and the proliferation of remote device/system management, the level of security at Internet-connected access points is critical. Therefore, the primary purpose of implementing a honeypot testbed is to analyze the penetration procedures and subsequent targets of attackers targeting industrial systems.

##### Lesson Learned, Future Work, and Limitations

The honeypot testbed had been deployed on a public IP address for about a month when publishing this article. During this period, there was a total of 1786 connection attempts where a large number of these connections were repeated attempts from the same IP addresses. Between these attempts, ten connections were identified that performed additional activity after a successful connection to the honeypot. With this solution, we were able to monitor the activities of attackers on industrial networks. It is crucial to create additional honeypots specialized for these networks to detect the areas and scopes in which the attackers are operating.

The main limitation of this testbed is its flexibility. The testbed is currently limited by the number of communication protocols and simulated devices. The source code allows both protocols and devices to be extensible. However, implementation is not entirely simple. In the case of adding a new device, it is necessary to implement its entire image so that a potential attacker does not get the impression that it is only a simulation.

### 3.3. Experience with Simulation Testbeds

#### 3.3.1. Brewery Testbed

The simulated testbed of the brewery is based on the physical testbed described in Section 3.1.3. The goal was to convert the physical testbed to perform the same processes as the physical testbed, containing as few elements as possible and not require a connection to physical sensors, switches, and other devices. We created this testbed in the second quarter of 2021 in connection with the physical testbed of the brewery. Based on this, the simulation was much easier due to the experience gained with the physical testbed.

##### Diagram of Brewery Testbed

**Figure 10:**
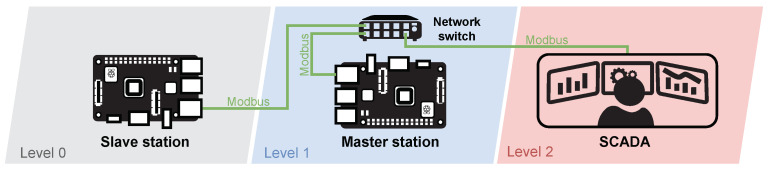
Diagram of brewery testbed.

As shown in Figure 10, the testbed consists of two Raspberry Pi 3B+ single-board computers (Level 0—Slave, Level 1—Master) and a remote monitoring application (Level 2—SCADA) of the installed server. Selected single-board computers ensure modularity and easy duplication of the entire testbed.

The software part of the testbed is based on the Raspbian operating system, which is a variant of Debian for Raspberry Pi devices. The simulation of all elements is provided by Python code with the pymodbus library, which is used to simulate Modbus protocol communication between slave, master and SCADA stations.

Initially, a simulated version of the brewery was designed to match the physical testbed. In the final version of the testbed, some parts of the process changed. The three-pot brewery was reduced to two-pot. Furthermore, automatic dosing of malt and hops has been added, which automates the whole process and does not require human intervention during production. Level indicators have been replaced by weight sensors that allow us to change the brewed volume; additionally, multi-directional valves for more efficient management have replaced binary valves. Due to this process update, it was possible to implement new devices and thus expand the variability of transmitted data. The changes can be seen in the scenario described below.

##### Components

The main goal of the simulated version of the brewery was to make it modular, implementable on as few elements as possible and at the same time mediate communication between at least two elements. As shown in Table 7, only four physical elements connected by network cables are required for implementation. The scenario itself can then be implemented using only two Raspberry Pi 3B+ with the software components installed. The SCADA element serves primarily as a display and a recording unit.

##### Scenario

**Figure 11:**
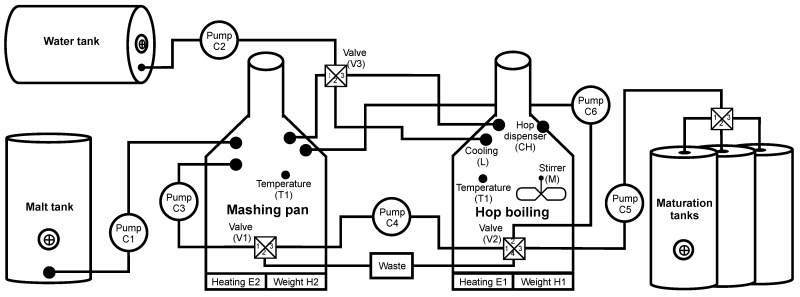
Diagram of the simulated brewery testbed.

The scenario simulating brewing beer on a Raspberry Pi is divided into five phases and is based on the diagram in Figure 11.

0. Rinsing—Before the cooking process, rinsing removes any chemical residue after cleaning. First, pump C2 is switched on (C2->ON). At the same time, valve V3 is switched to position 1 and the mashing pan is filled with water. During filling, the H2 sensor checks the weight to fill the required amount of water (H2->100l) and to prevent overflow of the container. When the water filling is completed, pump C3 is stopped and valve V3 is closed. Subsequently, pump C3 is switched on for 30 s, which flushes the pump and the mashing pan. The last part of the rinsing pan flush is switching the valve V1 to state 2, resulting in all the water being drained into the waste. In the second part, the hop boiling is rinsed. First, via pump C1 and valve V3 (switched to state 2), water is filled into the hop plant, where the sensor monitors the amount of filled water (H1->100l). Subsequently, 20 L of water are passed through pump C4 and valve V2->1; at the same time valve V1 switches to position 3 for filling water into the mashing pan. Finally, the exact process is performed via pump C5 (C5->ON), with valves V2 and V4 being switched to the required positions and water being pumped to the first fermentation tank (V2->4, V4->1). Then the water is discharged from the mashing pan and hop plant (V1->2, V2->4) into the waste.

1. Filling malt and heating water—Keeping the given temperatures is crucial to avoid unwanted tastes and aromas during the brewing process. In the first part, after pumping the malt, water is soaked and heated to 38 °C. Then, pump C1 is switched ON, and the defined amount of malt is pumped into the mashing pan. Next, pump C2 starts running, valve V3 is switched to state 3, and the hop boiling is filled with the defined amount of water. Furthermore, the heating of E1 to 3000 W is running until the temperature of 38 °C on T1 is reached. The last phase is the pumping of heated water from hop boiling to the mashing pan.

2. Mashing—The second main part of brewing beer is mashing, a process when sugars are extracted from malt. This operation is divided into several parts according to the cooking temperature.

a. During the the first phase of mashing, the temperature of 38 °C (T2 = 38 °C) is maintained in the mashing pan for 20 min. Next, stirring is switched on using pump C3 to prevent scorching and increase efficiency. After 20 min have elapsed, the temperature is raised to 52 °C (T2->52 °C) for 25 min.

b. During the second mashing phase (called the first mash), 2/3 of the volume is pumped from the mashing pan to the hop boiling (C4->ON, V1->3, V2->1), where the temperature is maintained at 52 °C (T1 = 52 °C). Subsequently, pump C3 is started (C1->ON, V1->2) and the mash temperature is raised to 62 °C (T2->62 °C) for 20 min. The temperature is then raised to 72 °C with a 20 min pause (T2 = 72 °C). After the pause, the mash is brought to a boil and boiled for 15 min. The remaining mash is then pumped into the hop boiling via a C4 pump (C4->ON, V1->3, V2->1). The thermometer T1 checks the temperature, which should be approximately 62 °C. If the temperature is lower, the heating to 62 °C is started (E1 = 1800 W). The last part of the first mash is pumping the entire 50 L volume from the hop boiling into the mashing pan.

c. The last stage of mashing is the second mash, which is very similar to the first. This part starts with two-thirds of the mash being pumped from the mashing pan into the hop boiling (C4->ON, V1->3, V2->1), maintaining a constant temperature of 62 °C (T1 = 62 °C). Next, in the mashing pan, the remaining part of the mash is heated to 72 °C min (T2->72 °C, E2 = 2000 W), where it is kept for 30 min. Then 1/3 of the mash is brought to the boil (T2 = 100 °C, E2 = 3000 W), maintained for 15 min. Contents of the mashing pan are then pumped into the hop boiling (C4->ON, V1->3, V2->1), where a temperature of 75 °C is reached by mixing the two parts of the mash. In case of a lower temperature, heating E1 is started. The last step of the second mash is pumping from the hop boiling to the mashing pan using pump C6 (C6->ON, V2->2).

3. Lautering—The mashing part is followed by lautering. Water is heated to 80 °C (E1 = 2000 W) in the hop boiling and then pumped to the mashing pan by the C6 pump (C6->ON, V2->2). The water flows over the solid parts of the mash and takes up the residual liquid parts, thereby increasing the malt yield.

4. Hop boiling—During the hop boiling process, the wort is pumped from the mash tun into the hop boiling (C1->3, C4->ON), where the contents are brought to the boil and held for 90 min (E1 = 3000 W, T1 = 100 °C). During this time, hops from the hop dispenser are added to the hop boiling at given intervals (CH->ON in 20/40/85 min). After the hop boiling is finished, the wort is cooled to 20 °C (L->ON, Stirrer->ON). After cooling, the wort is pumped into fermentation vessels (V2->3, C5->ON, V4->1).

0. Flushing—After the hopping process, the vessels are flushed with clean water and it begins the scenario.

##### The Purpose of the Testbed

Simulated environments have many advantages over physical environments. The user is not bound by physical elements in a simulated environment and can expand or duplicate parts as needed. There is also no risk of damage to individual elements, e.g., in the case of emulation of non-standard states. Simulated environments are thus suitable for various testing where physical elements or software parts, such as real PLCs, could be damaged. Additionally, due to their modularity, they are suitable, with advanced algorithm behaviour, as large volume generators.

##### Lesson Learned, Future Work, and Limitations

By implementing a simulated testbed of the brewery production, we were able to verify the functionality of the pyModbus library, which enables the implementation of Modbus protocol communication between two parties on the Ethernet link. The implemented testbed is also more suitable for various simulations in which irreversible damage or complex reconfiguration of the physical testbed would be threatened. The current focus is on the use of the testbed in simulating various attacks and its use/integration into the honeypot described in Section 3.2.

The main limitation of this testbed is its fidelity when the simulated devices do not reach the level of physical. For example, a simulated PLC unit only simulates an implemented scenario, but does not allow other possibilities of a real device. So it is not a full image of physical counterpart.

#### 3.3.2. Nuclear Power Plant Wastewater Treatment Testbed

The second simulated testbeds is similar to the physical testbed from Section 3.1.2. It also works with wastewater, but it simulates a collection tank in a nuclear power plant. Water is an essential part of nuclear power plants, used throughout the production cycle (e.g., in steam generators or cooling towers), resulting in wastewater that the plant has to treat. This part is simulated by the testbed described below.

We created this testbed in the first and second quarters of 2021. The implementation took place on the basis of information on infrastructure and communication within the part of the power plant dealing with wastewater recovery.

##### Diagram of Nuclear Power Plant Wastewater Treatment Testbed

**Figure 12:**
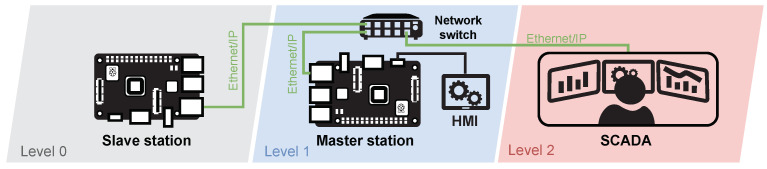
Diagram of the simulated wastewater treatment testbed.

As with the simulated brewery, the emphasis was on the number of elements. The testbed is implemented using two Raspberry Pi 3B+ (master/slave station), one network switch, a SCADA server and an HMI unit connected to the master station. Figure 12 shows the whole diagram embedded in the automation pyramid.

##### Components

As mentioned above, the testbed consists of two Raspberry Pi’s where the Raspbian 10 Buster operating system and the CPPPO library are installed to simulate the Ethernet/IP protocol. This protocol provides communication between the master, slave and SCADA stations. SCADA is implemented using the openMUC software solution. A touch screen LCD is also connected to the master station to allow local control of the scenario. All elements used within the testbed are listed in Table 8.

##### Scenario

**Figure 13:**
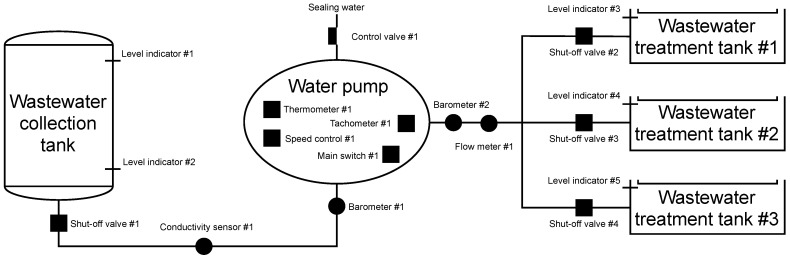
Scheme of wastewater treatment.

The scenario simulates a general model for pumping a wastewater collection tank at a nuclear power plant and can be seen in Figure 13. The tank is used to collect all water from the entire production unit. There are two water level indicators on the tank. Level indicator #1 is used to indicate if the tank is full. When the level in the tank reaches 900 cm, shut-off valve #1 opens, and control valve #1 opens to 20%. The water pump engine switches on and starts pumping. Level indicator #2 has the opposite function of level indicator #1. If the water level drops below 40 cm, the PLC signals to close the shut-off valve #1 and switches off the water pump motor. Level indicator #2 is in place because there are also solid particles in the wastewater that sediment at the bottom of the tank and form a sludge deposit, leading to clogging of the pump suction and equipment failure.

Indicators for measuring the levels of wastewater treatment tanks have a similar function. If the water in the wastewater treatment tank #1 reaches 1000 cm, the shut-off valve #2 closes, and then the control unit checks the level indicator #4. If it does not indicate that the tank’s level reaches 1000 cm, the shut-off valve #3 is open. After filling the wastewater treatment tank #2 (the level indicator indicates the value of 1000 cm), it continues to check the level indicator #5. If the wastewater treatment tank #3 is not complete, it will open the shut-off valve #4, and the tank fills. After filling all the wastewater treatment tanks, the shut-off valve #1 is closed, the pump motor is switched off, and an error message is displayed. Conductivity sensor #1 continuously measures conductivity to determine the level of radioactivity in the medium. If the conductivity value exceeds 330μ sieverts per square centimetre, shut-off valve #1 is closed, and the pump motor is switched off.

When the pump is running, a comparison of the pressure between suction and discharge is performed. In the case of different pressure, two situations can occur. In the first, the pressure difference may exceed the pressure added by the pump. This state is regulated by closing the control valve #1. The second option is to report that all wastewater treatment tanks are full, this option is unlikely in the simulation, but it can occur in actual operation. The pump adds pressure at 0.58 MPa, the line has a pressure of 0.4 MPa, so the maximum pressure value from barometer #1 is set as the barometer #2 + 0.58 MPa. If this value is exceeded, the shut-off valve #1 will be closed, and the pump motor will be stopped by the main switch #1.

Other important information is from thermometer #1. The temperature in the pump must not exceed 80 degrees Celsius. In nominal flow, the temperature in the pump is maintained at 60 °C. If the temperature exceeds 60 °C, the control valve #1 will open slightly by 10% because the sealing water has two uses. The primary purpose is to prevent the pumped liquid from leaking from the pump, the secondary purpose is to cool the rotating part of the pump, where the heat is generated by friction. The last flow parameter is monitoring information from tachometer #1, which provides information on the number of engine revolutions per second. The speed should be constant but an error may occur. The pump would give much higher pressure and could destroy pipes or other technologies. Therefore, there is a speed controller that keeps the speed at a nominal value of 2950 rpm.

##### The Purpose of the Testbed

The simulated testbed of wastewater treatment in nuclear power plant has some advantages over the physical one, as already described in The Purpose of the Testbed. The change from the previously simulated testbed is the used protocol, where the Ethernet/IP protocol is used instead of the Modbus protocol.

##### Lesson Learned, Future Work, and Limitations

By implementing a simulated testbed of a wastewater treatment plant in a nuclear power plant, we were able to verify the functionality of the CPPPO library, which enables the implementation of Ethernet/IP communication between two parties on an Ethernet link. As in the case of the previously simulated testbed of the brewery, this one is more suitable for simulating attacks or non-standard critical conditions that can cause damage to the PLC unit or end elements. As with the simulated brewery, we are currently focusing on implementing the testbed in the honeypot described in Section 3.2.

As in the case of the simulated brewery, the main limitation in this testbed is fidelity. Simulated devices do not reach the same level as physical ones. Simulation of the whole device would require considerable time and in some cases would make it impossible for flexibility, for example in the case of simulation of a new device.

### 3.4. Experience with Hybrid Testbeds

Hybrid testbeds are a combination of the previous testbeds, i.e., physical, simulation, and virtual. We decided to include emulated testbeds in this category, where individual physical devices (such as small computers) emulate real devices. There is usually an attempt to use at least some parts of the physical testbeds for hybrid testbeds, but this may not always be the case. These testbeds have advantages of great flexibility and scalability. They can therefore be used for multiple purposes and redesigned as needed. These testbeds have quite a lot of potential in the context of network communication research and analysis, where the environment can be more easily redesigned for different communication protocols and thus focus on the analysis of the protocol of interest.

#### 3.4.1. Industrial Production Loop

**Figure 14:**
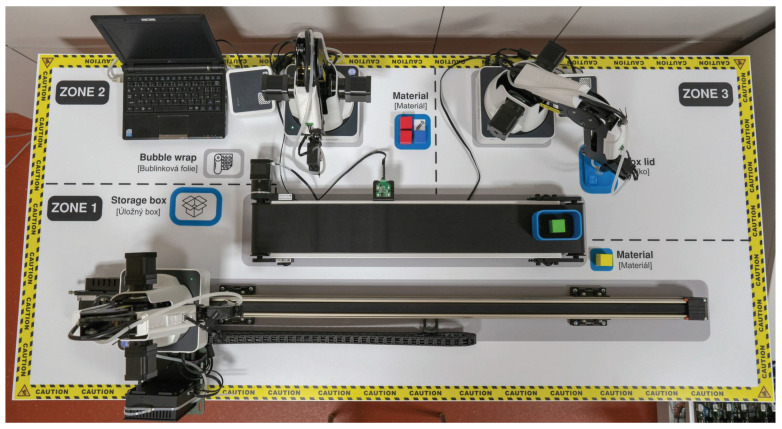
Industrial production loop.

This testbed simulates an industrial production loop where the main parts are industrial robotic arms. In this environment, control of the robotic arms was emulated using a Raspberry Pi device. The main goal was to create an environment in which different industrial communication protocols can be tested with the possibility of changing each protocol. Currently, this testbed uses Modbus-TCP and Ethernet/IP protocols for communication within a client-server architecture. The main reason for creating this testbed was its flexibility. Currently, the physical PLC S7-1200 is also implemented in this testbed, which will manage the communication with the individual Raspberry Pi and will control the entire operation. Testbed can be seen in the Figure 14. This testbed was created in June 2020. It is one of our first testbeds, and we are still optimizing it. The testbed is still active and still being used for data collection.

##### Diagram of Industrial Production Loop Testbed

**Figure 15:**
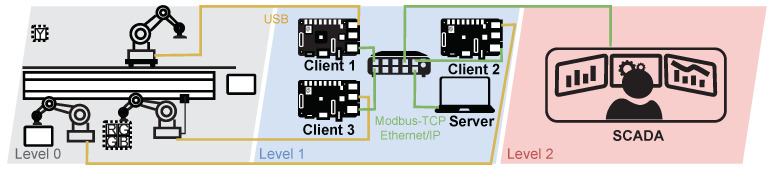
Diagram of industrial production loop.

A diagram of the wastewater treatment plant is depicted in Figure 15. Level 0 covers the physical process itself; three robotic arms work there and perform the packing and unpacking of the box. At level 2 there is the control process. Here, the three clients and the server communicate with the primary devices. Raspberry Pi simulates the clients; each is connected to one robotic arm via USB and controls their processes. Clients send a request to the server to see if it has any commands; communication with the server runs using Modbus-TCP or Ethernet/IP. We are currently implementing the control of this loop into our SCADA system based on OpenMUC. For now, it should communicate with this system using the Modbus-TCP protocol.

##### Components

Table 9 lists individual components of the testbed: hardware (devices and components), software, and protocols.

##### Scenario

**Figure 16:**
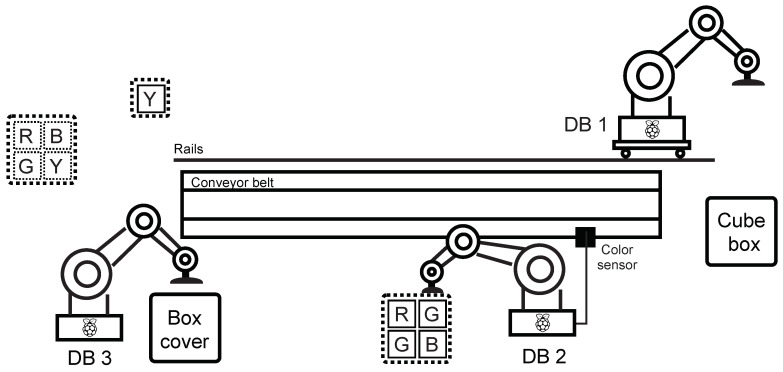
Scheme of production line.

In the production line scenario, a loop was created simulating a part of an industrial factory—in this case, an endless cycle of product assembly and subsequent decomposition. Dobot magician robotic arms and accessories—the Conveyor Belt Kit, which contains a colour sensor and a sliding rail kit, were used to implement the industrial loop. Furthermore, three Raspberry Pi in the 3B + version were used serving as a PLC for Dobot Magician communicating with the mater station (the fourth Raspberry Pi 3B +). All elements were connected using a network switch. The entire structure of the scenario is shown in Figure 16.

The general description of the industrial loop behaviour is divided into two parts—composition and decomposition (this creates an infinite cycle):

Composition:

- Step 1: Dobot DB1 picks up the box and places it on the belt. It then moves on the rail towards the end.
- Step 2: Dobot DB2 takes the filling and puts it in the box. Dobot DB3 then moves the belt with the box and padds to the colour sensor’s position.
- Step 3: Dobot DB2 takes one of the four cubes (randomly) and tests its colour with a colour sensor—if the colour is other than green, it returns the cube to its original position, if the colour of the cube is green, it puts it in the box. Next, Dobot DB3 moves the belt with the box to the end of the belt.
- Step 4: Dobot DB1 inserts the yellow cube in the box, Dobot DB3 puts the cover on the box—this completes the assembly cycle.

Decomposition:

- Step 1: Dobot DB3 removes the cover from the box and returns it to its original position, Dobot DB1 takes the yellow cube from the box and returns it to its original place. Next, Dobot DB1 moves to the starting position on the rail, Dobot DB3 moves the belt to the position of the colour sensor.
- Step 2: Dobot DB2 returns the green cube to the position from which it was taken, Dobot ID3 moves the box on the belt towards the start.
- Step 3: Dobot B2 returns the fill to its original position, Dobot DB1 returns the box to its original position—this completes the layout cycle.

##### The Purpose of the Testbed

The main reason for creating the testbed was the possibility of simple flexibility in terms of the used protocols. Currently, two industrial protocols can be selected on the testbed, namely Modbus-TCP and Ethernet/IP. The testbed is designed as before—for data capture of these protocols and subsequent communication analysis and use in detection systems. The advantage of this testbed is the Ethernet/IP protocol, which is quite popular, but the summary shows that it is not often included in testbeds. This testbed can also be used for testing various anomalies in industrial communication.

##### Lesson Learned, Future Work, and Limitations

Thanks to the construction of the testbed, we discovered the current possibilities of using libraries for industrial communication protocols. The Modbus-TCP library (pymodbus) and the Ethernet/IP library (cpppo) appeared to be the most valuable libraries in the design. Furthermore, we are currently testing the capabilities of the SNAP7 library, which allows communication with Siemens S7 PLCs. We are currently working on connecting a physical PLC to this testbed. This PLC should replace the server that the clients communicate with and should allow more accessible loop work. Sensors should also be connected to this PLC to move the testbed closer to real operations. This also implies the main limitation of this testbed, namely the lack of a physical PLC that would control the entire operation. This means inaccurate data compared to real operation.

### 3.5. Summary of Testbeds

For better clarity, we have created a summary of our testbeds. A comparison of the parameters of each testbed can be seen in Table 10. For the summarization, we based the parameters on Section 2. In the table we therefore give an overview of the components included in each testbed, then the characteristics of each testbed and finally the protocols used. It is important to note that our testbeds support SCADA systems and are gradually connected to our own SCADA system based on OpenMUC, where it is possible to communicate with devices using the Modbus-TCP protocol and in the testing phase we have implemented communication using the OPC UA protocol.

### 3.6. SCADA Software Based on OpenMUC

Management and control of industrial systems are administered from a central using SCADA software, which oversees all the equipment and data. There is a plethora of SCADA software ranging from specialized, for specific systems to proprietary ones focusing on one company’s products, to open-source ones that can handle many protocols. The advantage of open-source solutions is also their disadvantage. The openness leads to great modularity to devices from different manufacturers. Still, their support and up-to-dateness are usually insufficient to the rest of the systems. When selecting a suitable SCADA software, we had specific criteria set: modularity, free license, open-source, easy implementation of additional protocols, and functionality in a web browser. We compared Free SCADA 2, Indigo SCADA, openDAX, S.E.E.R, 2, openSCADA, mySCADA, PROMOTIC, and openMUC. However, most of the software is outdated, does not support the implementation of additional protocols, or is not open source. Therefore, the only candidate that meets all the criteria while working with large industrial companies is openMUC.

The OpenMUC system is based on the OSGi Java framework and is primarily designed to facilitate the development of specialized monitoring, control and logging systems. The possibility of creating simple logging applications as well as complex SCADA systems seem a great advantage. Because the system is developed under the auspices of the Fraunhofer Institute, it also has a good foundation and relatively strong support from both the community and the developers themselves. Its functionality has been tested on several projects under the auspices of the institute mentioned above. Another significant asset is the available interfaces and drivers for the following protocols: Modbus TCP/Modbus RTU/TCP, KNX, M-Bus (wired), and wireless M-Bus. Other protocols can be easily implemented and added to the OSGi package. Besides the advantages mentioned above, the ability to run on most operating systems (thanks to the JRE) and the web interface is also very convenient.

## 4. Discussion and Results

This article focused on the current state of individual industry testbeds, deliberately leaving out testbeds in the energy sector and focusing mostly on the manufacturing, food, and process industry. In the paper, we have demonstrated our testbed design approaches and commented on these approaches in relation to the testbed summary table.

**Figure 17:**
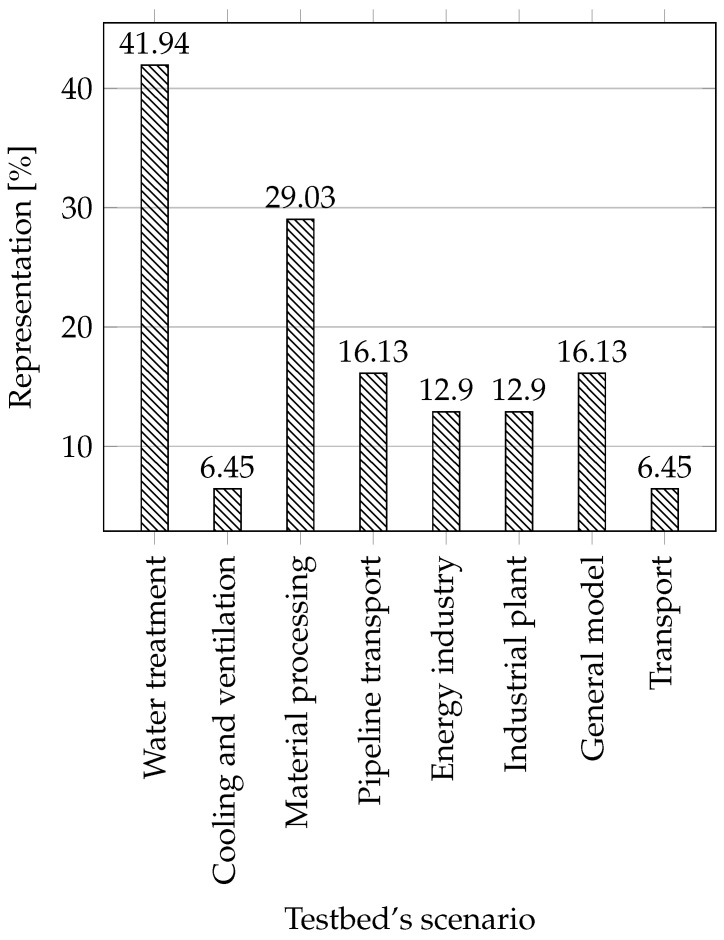
The percentage of selected testbeds.

According to Figure 17, our summary shows that most testbeds are focused on the area of water treatment at 41.94%. This is due to the relatively easy construction of such testbeds and their rather high level of realism. The SWAT testbed [43] also plays a role in this representation, in our summary it has received the highest level of confidence and it is generally referenced in a large number of papers. The testbed from the production line and material processing domain has the second-highest representation, with 29.03%. In percentage, the other areas of testbeds are represented somewhat similarly.

**Figure 18:**
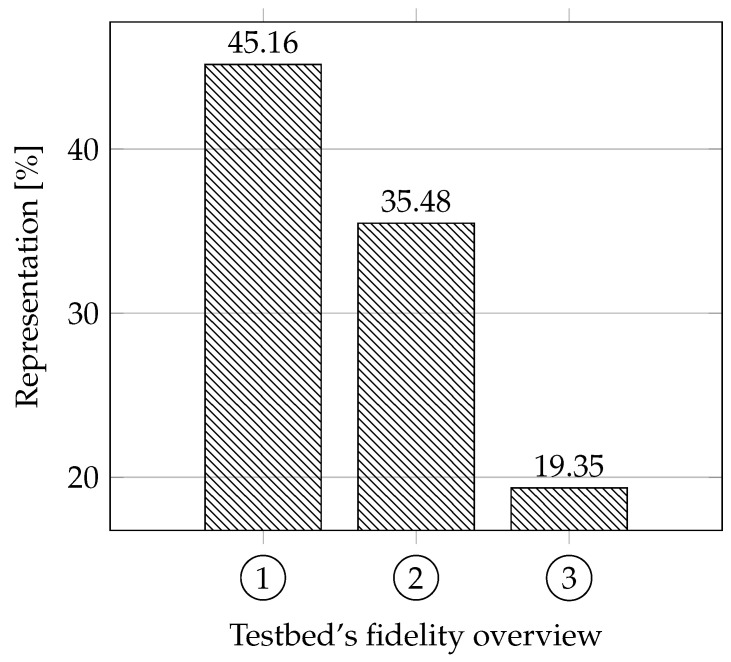
The percentage of selected testbeds’ fidelity.

We then compared percentages by the level of confidentiality. The comparison can be seen in Figure 18. As expected, the highest number of testbeds with confidence level 1 is 45.16%. Testbeds with a trust level of 35.48% follow, the smallest number of testbeds with a maximum trust level of 19.35% (i.e., six testbeds meet our conditions) are presented in last place. patterns

**Figure 19:**
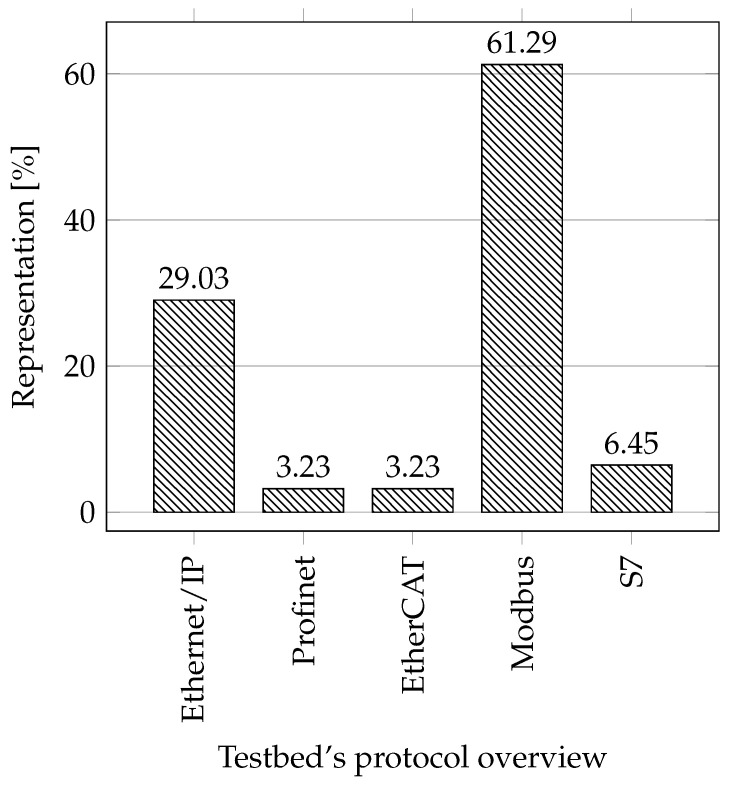
The percentage of selected testbeds’ protocols.

We have also included industrial protocols like Ethernet/IP, Profinet EtherCAT Modbus, and the proprietary Siemens S7 protocol in our summary. From the graph in Figure 19, we can see that the Modbus protocol has the most significant representation with 61.29%. This is due to the fact that it is one of the oldest industrial protocols, but also thanks to its easy implementation and available libraries. The second most represented industrial protocol in these testbeds is Ethernet/IP with 29.03%. Other protocols are very sparely represented. Since Profinet has currently the largest market presence, according to HMS Networks [61] research, there is a need to create more testbeds dedicated to this protocol. It is represented only once in the testbed summary. It is also worth focusing on EtherCAT, which has already surpassed Modbus in deployment but is only represented once in the testbeds. Siemens’ significant market share makes it worth looking at their proprietary S7 protocol. We tried to focus on Profinet, S7 and Ethernet/IP protocols in our testbeds due to such a small representation. It was quite problematic to focus on the EtherCAT protocol as we have not found suitable libraries to implement the communication of this protocol to, and currently, we do not have any devices supporting this communication.

**Figure 20:**
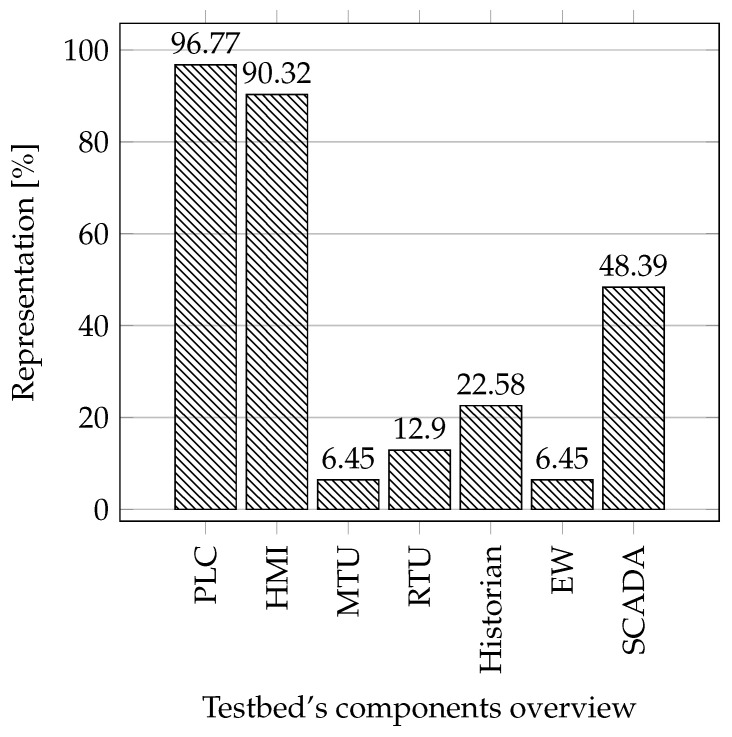
The percentage of selected testbeds’ components.

We have also focused on single industrial components; in summary, the graph in Figure 20 shows the percentage of each component in the testbeds summary. From the summary, it can be seen that almost all testbeds contain the essential component—PLC, i.e., 96.77% (for one testbed, it was not possible to determine whether it includes PLC). The second vital component is the HMI, which 90.32% of the testbeds contain. The rest of the components is represented in the testbeds only to a minimal amount. We have also added the percentage of testbeds that include specialized SCADA software for remote control of the environment to this graphical overview; almost half of these testbeds (48.39%) meets this requirement.

**Figure 21:**
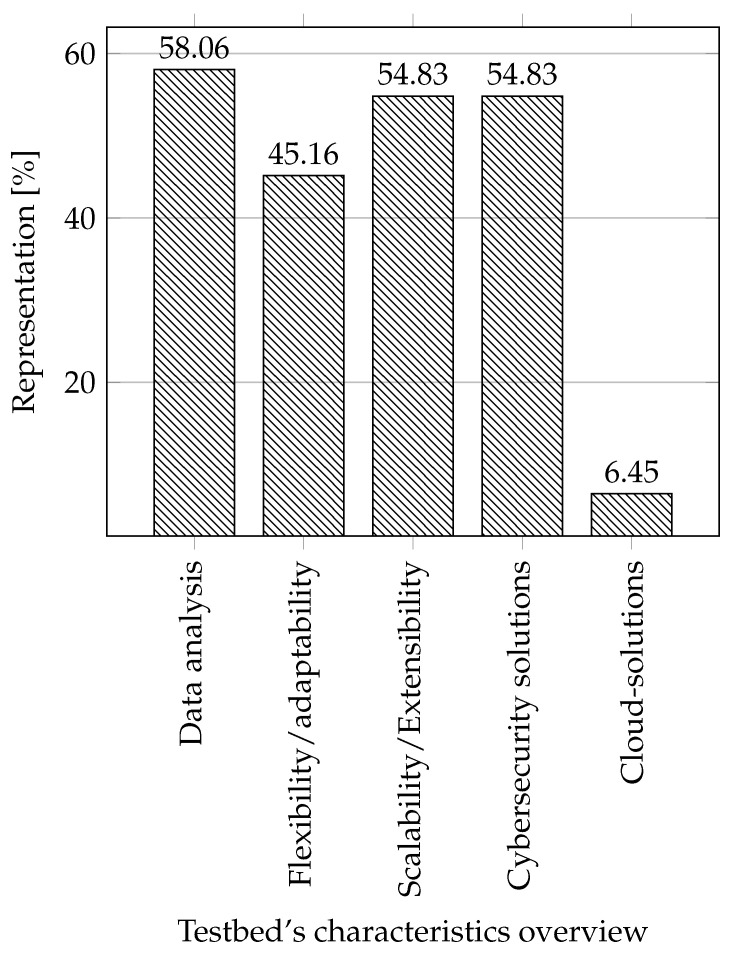
The percentage of selected testbeds’ characteristics.

The last graph in Figure 21 compares the percentages of testbed characteristics. These are the characteristics, properties, or focus of each testbed. Here, the representation is fairly even, with most testbeds being used for data acquisition or working with data (58.06%). A very small number of testbeds (6.45%, i.e., two) are focused on cloud solutions. In this point, we can see further potential and challenges for research.

Our work shows sample space for creating individual test environments. Currently, there are many solutions, but most of them only touch individual parts of industrial networks superficially. Creating individual testbeds will help understand theoretical parts in industrial security, especially in areas of communication protocols and their security, and the security of individual physical components such as PLC, HMI, MTU, and more. For research in cybersecurity areas, this can mean a greater understanding of the functionalities of real devices and a complete understanding of entire SCADA systems. From a practical point of view, we need to create more testbeds with higher fidelity values which advantages are already by name in the complete emulation of real systems. Testbeds with a fidelity value of 3 will help in research for better results. The contribution of this survey is to show the current status and limitations in testbeds for possible research in ICS networks. The disadvantage of these solutions is that they are only a simulation of real-life processes. It is not easy to include the entire industrial network in the testbed due to the amount of equipment used in actual operations. Due to the human factor, one of the main issues in cybersecurity and within the ICS, there may be employees not trained in this area. patterns patterns

### Open Research Challenges

With this visualized summary, we can define challenges for further research in testbeds for industrial networks. We can see an excellent expanse for more effective implementation of protocols different than Modbus-TCP, especially for Profinet and EtherCAT. OPC protocol is increasingly used for communication within SCADA systems with lower layer devices; this protocol was not present in any of the testbeds. Other challenges also arise in the creation of more complex testbeds in terms of the components used. Most testbeds are created only on implementing PLC and HMI and lack other essential components that appear in real solutions; this would also help increase the credibility of the individual testbeds. These individual extensions are particularly suitable for collecting data that will lead to improved detection systems. Thus, one of the main directions in research at present is the creation of suitable anomaly detection systems for ICS. Such improved testbeds in the future can help us to more extensive detection and improved results. It also makes more training data for machine learning based detection systems. Finally, there seems to be a relatively large field for research in the area of cloud solutions and the implementation of industrial components in these solutions.

## 5. Conclusions

In this article, we first discussed the importance of creating testbeds for industrial control systems, primarily from a cybersecurity and data generation point of view. We also covered a general description of the testbeds applicability in this area of interest. Furthermore, we provided an overview of the current state of the art in the field of industrial testbeds, where we covered all industrial domains outside the power industry. Based on other summary articles, we derived and modified a methodology for comparing individual parameters for testbeds. Based on this methodology, we have created summary tables to compare the parameters of 31 testbeds. Furthermore, we described our own experience in creating physical, simulated, virtual, and hybrid testbeds. For each approach, we have demonstrated our own solution and described how we approached the creation, what we learned through the testbed and what is planned for the future with testbeds. We also justified using our software for SCADA systems based on the open-source solution OpenMUC, which we used in some testbeds. Finally, we visualized the percentage of each parameter from the testbed summary, which allowed us to define potential challenges for further research in this area. This comparison allowed us to consider some aspects when building further testbeds, especially in terms of the industrial protocols used and the implementation of the individual components.

The limitations of our work are mainly in the layer representation of the individual testbeds. The testbeds include carefully selected applications that represent specific parts in the industrial process and environments. We focus right now mainly on lower levels of the industrial pyramid. Thanks to the fully live operations, there are no limitations for connecting it with higher levels, including applications such as MES/MOM, CAD/CAE/CAM or ERP/CRM, and more. This approach might be seen as part of future research, as it brings another layer to Industry 4.0.

## Figures and Tables

**Table 1:** Testbed overview.

			Scenario									Level			Protocols					Usecase			
ID	Year	Inst.	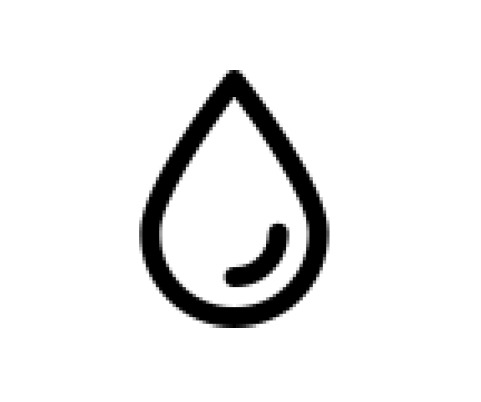	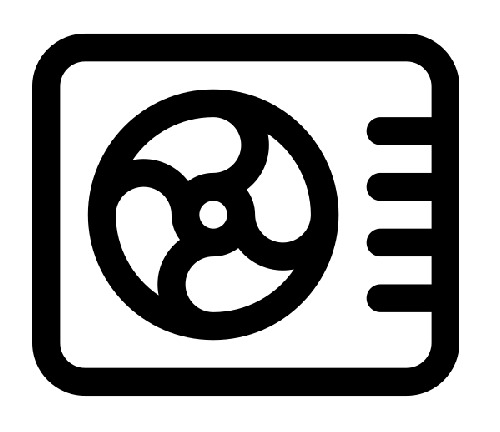	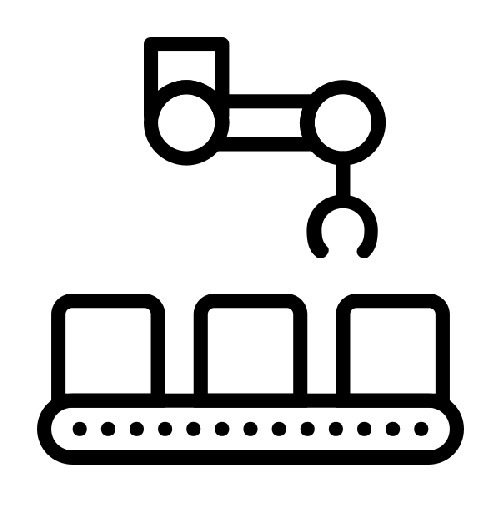	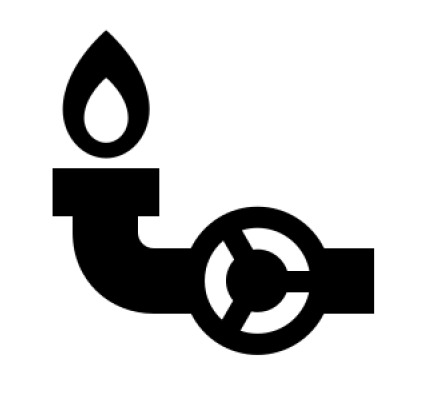	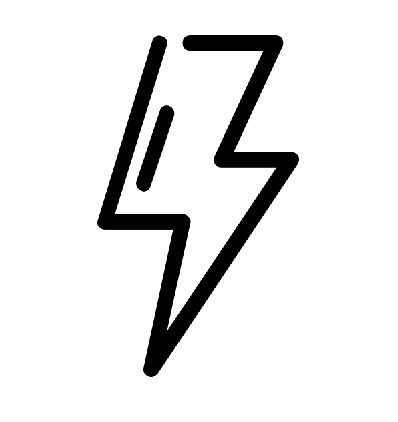	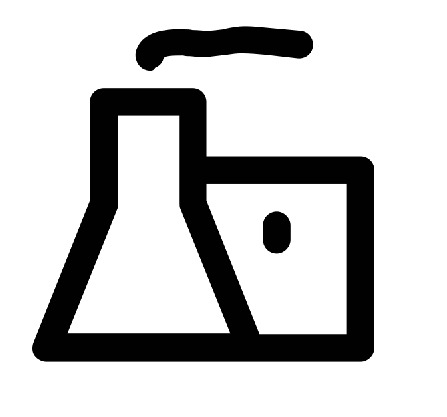	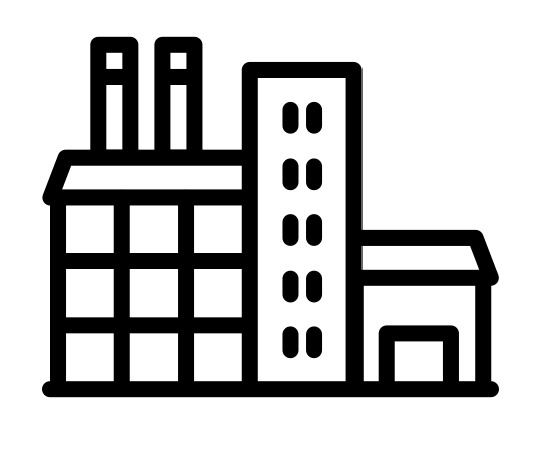	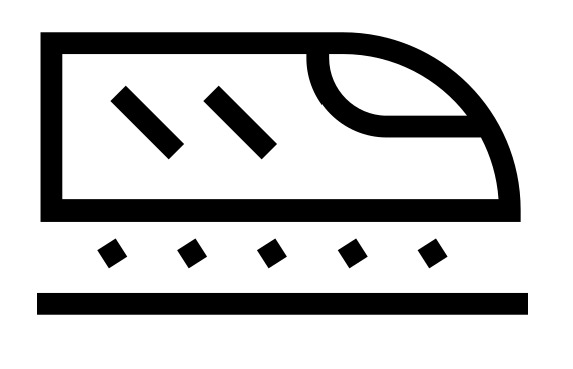	Fid.	0	1	2	Ethernet/IP	Profinet	EtherCAT	Modbus	S7	Education	Cybersec	SCADA	Reference
1	2011	MSU	✓	✓	✓	✓	✓	✕	✕	✕	②	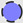	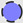	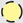	✓	✕	✕	✓	✕	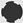	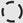	✓	[30]
2	2011	JRC	✕	✕	✕	✕	✕	✓	✕	✕	①	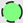	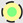	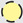	✕	✕	✕	✓	✕	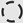	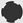	✓	[31]
3	2012	SNL	?	?	?	?	?	?	?	?	③	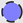	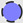	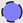	✕	✕	✕	✓	✕	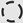	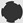	✓	[32]
4	2013	QUT	✓	✕	✓	✓	✓	✕	✕	✕	①	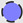	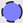	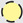	?	?	?	?	?	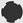	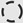	✓	[33]
5	2013	RMIT	✓	✕	✕	✕	✕	✕	✕	✕	①	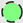	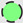	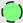	✕	✕	✕	✓	✕	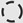	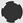	✓	[34]
6	2013	CNITSEC	✕	✕	✕	✕	✕	✕	✓	✕	①	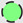	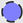	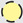	?	?	?	?	?	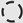	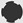	✕	[35]
7	2013	AUB	✕	✕	✕	✕	✕	✕	✓	✕	①	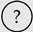	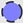	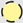	✕	✕	✕	✕	✕	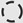	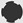	✕	[36]
8	2014	NIST	✕	✕	✓	✕	✕	✓	✕	✕	①	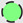	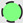	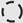	✓	✕	✓	✕	✕	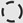	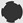	✕	[37]
9	2014	TU	✕	✕	✕	✕	✕	✕	✓	✕	②	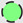	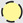	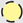	✕	✕	✕	✓	✕	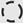	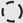	✓	[38]
10	2016	UNO	✓	✕	✕	✓	✓	✕	✕	✕	②	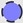	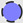	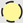	✓	✓	✕	✓	✕	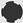	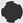	✕	[39]
11	2016	UAH	✕	✕	✕	✓	✕	✕	✕	✕	②	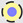	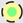	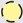	✕	✕	✕	✓	✕	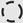	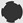	✕	[40]
12	2016	KFUPM	✓	✕	✕	✕	✕	✕	✕	✕	②	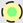	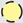	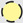	✕	✕	✕	✓	✕	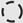	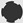	✓	[41]
13	2016	AU	✓	✕	✕	✕	✕	✕	✕	✕	①	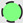	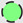	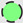	✕	✕	✕	✓	✕	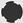	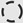	✕	[42]
14	2016	UTD, SUTD	✓	✕	✕	✕	✕	✕	✕	✕	③	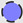	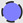	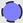	✓	✕	✕	✕	✕	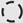	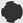	✓	[43]
15	2016	UCBMR, URT	✓	✕	✕	✕	✕	✕	✕	✕	②	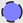	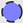	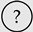	✕	✕	✕	✓	✕	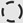	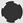	✕	[44]
16	2017	IIC	✕	✕	✓	✕	✕	✕	✕	✕	③	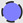	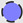	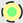	✕	✕	✕	✓	✕	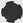	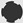	?	[45]
17	2017	SUTD	✓	✕	✕	✕	✕	✕	✕	✕	③	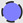	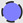	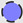	✓	✕	✕	✕	✕	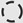	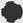	✓	[46]
18	2018	IFET	✓	✕	✕	✕	✕	✕	✕	✕	①	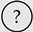	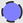	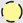	✕	✕	✕	✓	✕	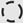	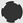	✕	[47]
19	2018	CSIT	✓	✕	✕	✓	✕	✕	✕	✕	①	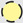	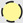	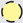	✕	✕	✕	✕	✕	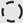	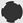	✕	[48]
20	2018	SKLMEAC, ZU	✕	✕	✕	✕	✕	✓	✕	✕	②	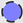	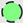	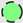	✕	✕	✕	✕	✕	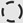	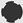	✕	[49]
21	2018	HS	✕	✕	✕	✕	✕	✕	✓	✕	①	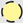	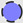	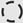	✓	✕	✕	✓	✓	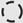	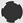	✕	[50]
22	2019	CAS	✕	✕	✓	✕	✓	✓	✕	✓	②	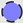	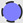	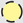	✓	✕	✕	✓	✓	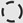	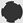	✕	[51]
23	2019	HS	✕	✕	✓	✕	✕	✕	✕	✕	①	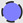	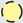	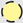	✕	✕	✕	✓	✕	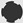	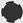	✓	[52]
24	2019	UT, NIT	✓	✕	✕	✕	✕	✕	✕	✕	②	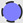	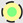	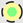	✕	✕	✕	✕	✕	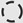	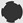	✓	[53]
25	2019	SHSU	✕	✕	✓	✕	✕	✕	✕	✕	②	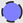	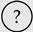	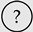	✕	✕	✕	✓	✕	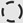	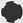	✓	[54]
26	2019	IIRS	✕	✕	✕	✕	✕	✕	✕	✓	③	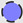	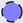	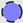	✕	✕	✕	✓	✕	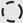	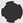	✕	[55]
27	2019	BCS	✓	✕	✓	✕	✕	✕	✕	✕	②	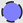	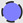	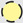	?	?	?	?	?	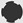	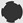	✓	[56]
28	2020	ORNL	✕	✓	✕	✕	✕	✕	✕	✕	③	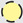	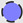	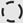	✓	✕	✕	✕	✕	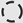	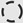	✕	[57]
29	2020	BU, OPNZ, SQU	?	?	?	?	?	?	?	?	①	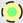	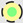	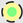	✕	✕	✕	✓	✕	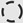	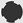	✓	[58]
30	2020	UEC	✕	✕	✓	✕	✕	✕	✕	✕	①	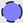	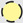	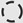	✓	✕	✕	✓	✕	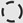	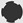	✕	[59]
31	2020	MU	✕	✕	✕	✕	✕	✕	✓	✕	①	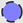	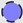	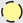	?	?	?	?	?	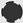	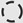	✓	[60]

**Table 2:** Testbeds’ parameters.

ID	1	2	3	4	5	6	7	8	9	10	11	12	13	14	15	16	17	18	19	20	21	22	23	24	25	26	27	28	29	30	31
PLC	✓	✓	✓	✓	✓	✓	✓	✓	✓	✓	✓	✓	✓	✓	✓	?	✓	✓	✓	✓	✓	✓	✓	✓	✓	✓	✓	✓	✓	✓	✓
HMI	✓	✕	✓	✓	✓	✓	✓	✓	✕	✓	✓	✓	✓	✓	✓	?	✓	✓	✓	✓	✓	✓	✓	✓	✓	✓	✓	✓	✓	✓	✓
MTU	✓	✕	✕	✕	✓	✕	✕	✕	✕	✕	✕	✕	✕	✕	✕	?	✕	✕	✕	✕	✕	✕	✕	✕	✕	✕	✕	✕	✕	✕	✕
RTU	✓	✕	✕	✕	✕	✓	✕	✕	✓	✕	✕	✕	✕	✕	✕	?	✓	✕	✕	✕	✕	✕	✕	✕	✕	✕	✕	✕	✕	✕	✕
Historian	✕	✕	✕	✕	✕	✕	✕	✓	✕	✕	✕	✕	✕	✕	✕	?	✓	✓	✓	✕	✕	✕	✓	✕	✓	✕	✕	✓	✕	✕	✕
Engineering Workstation	✕	✕	✕	✕	✕	✓	✕	✕	✕	✕	✕	✕	✕	✕	✕	?	✓	✕	✕	✕	✕	✕	✕	✕	✕	✕	✕	✕	✕	✕	✕
Data analysis	✓	?	✓	✕	✓	?	✓	✓	✓	✕	✓	✕	?	✓	✓	?	✓	✓	✓	?	✓	✕	✕	✓	✓	✓	✓	✓	✕	✕	✕
Flexibility/ adaptability	✕	✕	✓	✕	✕	✕	✕	?	✕	✕	✕	✓	✓	✓	✓	✓	✕	✕	✓	✓	✓	✓	✕	✓	✓	✓	✕	✕	?	✕	✓
Scalability/ Extensibility	?	✓	✓	✕	✓	✓	✕	✕	✕	✕	✕	?	✕	✓	✓	✓	✕	✕	✓	✓	✓	✓	✕	✓	✓	✓	✓	✓	✕	✕	✓
Cybersecurity solutions	?	✓	✓	✓	✓	?	?	✓	✓	✕	✕	✓	✓	✓	✓	✓	✕	✕	✕	?	✕	✕	✕	✓	✓	✓	✓	✓	✓	✕	✕
Cloud-solutions	✕	✕	✕	✕	✕	✕	✕	✕	✕	✕	✕	✕	✕	✕	✕	✕	✕	✕	✕	✕	✕	✕	✕	✓	✕	✓	✕	✕	✕	✕	✕

**Table 3:** Components of industrial motor control system testbed.

Hardware Name	Hardware Description	Software		Protocols
Siemens s7-1200	PLC	TIA portal v15.1	Programming Supervision	Profinet
Siemens KTP700	HMI			S7comm
Siemens Scalance XB005	Industrial Switch			
Siemens V90	Driver			
Simotics S-1FL6	Servomotor			
Computer	For industrial software			

**Table 4:** Components of wastewater treatment testbed.

Hardware Name	Hardware Description	Software	
Siemens s7-1200	PLC	TIA portal v15.1	Programming Supervision
Siemens KTP700	HMI		
Siemens CSM1277	Industrial Switch		
Submersible pumps	Binary pumps	**Protocols**	
Non-contact liquid level sensors	Binary sensors	Profinet	
Vertical water level sensors	Binary sensors	S7comm	
Float switches	Binary sensors		
Water pumps	Binary pumps		
Water pumps as a blowers	Binary pumps		
Computer	For industrial software		

**Table 5:** Components of physical brewery testbed.

Hardware Name	Hardware Description	Protocols		
Siemens s7-300	PLC	Profinet		
Siemens KTP700	HMI	S7comm		
Siemens XB005	Industrial Switch			
Valves	Binary valves for flow control		**Software name**	**Software description**
Relays	Binary relay—heating, cooling, stirred, pump	TIA portal v15.1	Programing Supervision	
Level indicator	Binary float switches for level monitoring	openMUC	SCADA interface	
Thermometers	Analog thermometer for temperature monitoring			
Barometer	Analog barometer for pressure monitoring			

**Table 6:** Components of honeypot testbed.

Software Name	Software Decription	Protocols
HonSSH	Honeypot for SSH protocol	ModBus
MiniNet	Network infrastructure simulator	Ethernet/IP
MiniCPS	Industrial systems simulator	SSH
Docker	Separation of the honeypot from the rest of the OS	
Suricata	Honeypot intrusion logging	**Hardware**
Grafana	Monitoring of all honeypot elements	Server with public IP adress
Ubuntu VPS	Operating system for honeypot virtualization	

**Table 7:** Components of the simulated brewery testbed.

Software Name	Software Description	Protocols	Hardware
Rasbian 10 Buster	Operating system for RP	ModBusTCP	Raspberry Pi 3B+
pymodbus 2.5.2	Modbus library in python		Network switch
openMUC	SCADA		

**Table 8:** Components of the simulated wastewater treatment testbed.

Software Name	Software Decription	Hardware	Protocols
Rasbian 10 Buster	Operating system for RPi	Raspberry Pi 3B+	Ethernet/IP
CPPPO	Modbus library in python	Network switch	
openMUC	SCADA	Touch LCD display	

**Table 9:** Components of industrial production loop testbed.

Hardware Name	Hardware Description	Software	
Dobot Magician	Robotic Arm 3×	Python scripts	For clients and server
Raspberry Pi 3B+	Client for control robotic arm 3×	OpenMUC	As a SCADA
Computer	Server for managing the communication		
Dobot Conveyro belt	To move the material	**Protocols**	
Dobot Sliding Rail	To move the dobot	Modbus–TCP	
Color sensor	To select the right material	Ethernet/IP	

**Table 10:** Summary of testbeds.

		Industrial Motor Control System	Wastewater Treatment	Physical Brewery	Simulation Brewery	Power Plant Waste-Water Treatment	Industrial Production Loop	Honeypot
**Components**	**PLC**	✓	✓	✓	✓	✓	✓	✕
	**HMI**	✓	✓	✓	✕	✓	✓	✕
	**MTU**	✓	✓	✓	✓	✕	✕	✕
	**RTU**	✕	✕	✕	✕	✕	✕	✕
	**Historian**	✕	✕	✕	✕	✕	✕	✕
	**Engineering workstation**	✓	✓	✓	✕	✕	✕	✕
**Characteristic of testbeds**	**Data analysis**	✓	✓	✓	✓	✓	✓	✓
	**Cybersecurity**	✓	✓	✓	✓	✓	✓	✓
	**Cloud-solution**	✕	✕	✕	✕	✕	✕	✕
	**Education**	✓	✓	✓	✓	✓	✓	✓
	**Flexibility/adaptability**	✕	✕	✕	✓	✕	✕	✓
	**Scalability/Extensibility**	✕	✕	✕	✓	✓	✓	✓
	**Fidelity**	✓	✓	✓	✕	✕	✕	✓
**Protocols**	**Profinet**	✓	✓	✓	✕	✕	✕	✕
	**Ethernet/IP**	✓	✓	✓	✕	✓	✓	✓
	**EtherCAT**	✕	✕	✕	✕	✕	✕	✕
	**ModBus-TCP**	✕	✕	✕	✓	✕	✓	✓
	**S7**	✓	✓	✓	✕	✕	✕	✕

## Abbreviations

The following abbreviations are used in this manuscript:

CTF	Capture the flag
CAD	Computer-aided design
CAE	Computer-aided engineering
CAM	Computer-aided manufacturing
CRM	Customer relationship management
DCS	Distributed control system
EW	Engineering workstation
ERP	Enterprise resource planning
HMI	Human–machine interface
ICS	Industrial control system
IT	Information technologies
IaaS	Infrastructure as a service
IDS	Intrusion detection system
LHMI	Local HMI
MES	Manufacturing execution system
MOM	Manufacturing operations management
MTU	Master terminal unit
OT	Operational technologies
PLC	Programmable logic controller
RTU	Remote terminal unit
SBR	Sequencing batch reactor
SCADA	Supervisory control and data acquisition
